# Unc-51/ATG1 Controls Axonal and Dendritic Development *via* Kinesin-Mediated Vesicle Transport in the *Drosophila* Brain

**DOI:** 10.1371/journal.pone.0019632

**Published:** 2011-05-12

**Authors:** Hiroaki Mochizuki, Hirofumi Toda, Mai Ando, Mitsuhiko Kurusu, Toshifumi Tomoda, Katsuo Furukubo-Tokunaga

**Affiliations:** 1 Graduate School of Life and Environmental Sciences, University of Tsukuba, Tsukuba, Japan; 2 Structural Biology Center, National Institute of Genetics, and Department of Genetics, The Graduate University for Advanced Studies, Shizuoka, Japan; 3 Division of Neurosciences, Beckman Research Institute of the City of Hope, Duarte, California, United States of America; Columbia University, United States of America

## Abstract

**Background:**

Members of the evolutionary conserved Ser/Thr kinase Unc-51 family are key regulatory proteins that control neural development in both vertebrates and invertebrates. Previous studies have suggested diverse functions for the Unc-51 protein, including axonal elongation, growth cone guidance, and synaptic vesicle transport.

**Methodology/Principal Findings:**

In this work, we have investigated the functional significance of Unc-51-mediated vesicle transport in the development of complex brain structures in *Drosophila*. We show that Unc-51 preferentially accumulates in newly elongating axons of the mushroom body, a center of olfactory learning in flies. Mutations in *unc-51* cause disintegration of the core of the developing mushroom body, with mislocalization of Fasciclin II (Fas II), an IgG-family cell adhesion molecule important for axonal guidance and fasciculation. In *unc-51* mutants, Fas II accumulates in the cell bodies, calyx, and the proximal peduncle. Furthermore, we show that mutations in *unc-51* cause aberrant overshooting of dendrites in the mushroom body and the antennal lobe. Loss of *unc-51* function leads to marked accumulation of Rab5 and Golgi components, whereas the localization of dendrite-specific proteins, such as Down syndrome cell adhesion molecule (DSCAM) and No distributive disjunction (Nod), remains unaltered. Genetic analyses of kinesin light chain (*Klc*) and *unc-51* double heterozygotes suggest the importance of kinesin-mediated membrane transport for axonal and dendritic development. Moreover, our data demonstrate that loss of *Klc* activity causes similar axonal and dendritic defects in mushroom body neurons, recapitulating the salient feature of the developmental abnormalities caused by *unc-51* mutations.

**Conclusions/Significance:**

Unc-51 plays pivotal roles in the axonal and dendritic development of the *Drosophila* brain. Unc-51-mediated membrane vesicle transport is important in targeted localization of guidance molecules and organelles that regulate elongation and compartmentalization of developing neurons.

## Introduction

Neurons are highly polarized and compartmentalized cells with an extended axon and highly branched dendrites. Apart from their role in propagating electrical signals, axons serve as a track for long-distance transport of synaptic components that are synthesized in the soma and delivered to the nerve terminals [1]. In mature neurons, membrane trafficking and cargo delivery are essential for their dynamic physiological functions, which depend on active transport of synaptic vesicles [1]. Membrane trafficking is also essential in developing neurons for delivering diverse organelles and molecules that are required for elongation and guidance of the growing axonal and dendritic termini [1], [2], [3], [4], [5], [6]. Given the elaborate internal compartments, efficient and controlled membrane vesicle trafficking in neuronal cells is thought to be critical in brain development to establish the functional circuitry [7], [8], [9], [10].

Members of the conserved Ser/Thr kinase Unc-51 family (Unc-51/Unc51.1/Unc51.2) are key regulatory proteins that control axonal elongation during nervous system development in *C. elegans* and mice [11], [12], [13], [14], [15], [16]. In mouse, Unc51.1 and Unc51.2 are expressed in a number of neuronal populations during development, including cerebellar granule cells and spinal sensory neurons, in which these proteins are localized to vesicular structures in growth cones [13], [14], [17]. Molecular studies have shown that Unc-51 controls axon formation in the granule cells through the endocytic membrane trafficking pathway *via* directly binding the synaptic GTPase activation protein (SynGAP) and Syntenin, which is a PDZ domain-containing scaffolding protein that binds Rab5 GTPase and is involved in endocytic vesicular turnover [14]. In *C. elegans*, *unc-51* mutants exhibit premature axonal termination, abnormal trajectories and extra-axon branches with abnormal vesicles and the accumulation of cisternae, suggesting underlying defects in membrane vesicle trafficking [12], [18]. Unc-51 interacts with Vab-8, which contains a kinesin motor-like domain, to control axon extension through regulation of the Netrin receptor Unc-40/DCC and the Slit receptor SAX-3/ROBO [19], [20], [21]. Studies in *C. elegans* have also shown that Unc-51 interacts with Unc-14, a RUN domain protein, which regulates kinesin 1-dependent vesicle transport by binding to Unc-16/JIP3/JSAP1, a cargo adaptor for the kinesin motor proteins [22], [23]. In addition, recent studies in *Drosophila* have shown that Unc-51 regulates the association of synaptic vesicles and motor proteins *via* interacting with Unc-76, a kinesin heavy chain (Khc) adaptor protein [16]. Unc-51 phosphorylates Unc-76, which then interacts with Synaptotagmin 1 (Syt 1), a synaptic vesicle protein. Collectively, these lines of evidence suggest that Unc-51 regulates the trafficking of early endosomes and their molecular cargos in diverse neuronal cells.

In this work, we investigated the functional significance of Unc-51 in neural development, focusing on its regulatory functions in kinesin-dependent vesicle transport in the developing *Drosophila* brain. In the larval mushroom bodies (MBs), which are centers of olfactory associative learning [24], [25], [26], [27], [28], Unc-51 is preferentially expressed in newly elongating axons. Mutations of *unc-51* cause disintegration of axonal bundles with aberrant accumulation and mislocalization of Fasciclin II (Fas II), an IgG type cell adhesion/guidance molecule. By genetic interaction experiments, we show that *unc-51* interacts with the kinesin light chain (*Klc*) gene in MB development, and that *Klc* mutation causes dendritic and axonal defects that are reminiscent of *unc-51* mutants. These results demonstrate the pivotal role of Unc-51 in axonal and dendritic development in the brain, and highlight the conserved functions of Unc-51 in the trafficking of diverse membrane vesicles and cargo molecules that are essential to the growth and guidance of developing neurons.

## Results

### Organization of the *Drosophila* larval brain

**Figure 1:**
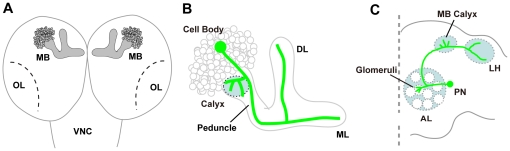
Schematic representations of the larval MB and the adult olfactory PNs. (A) The *Drosophila* larval brain. MB, mushroom body; OL, optic lobe primordium; VNC, ventral nerve cord. (B) Structure of the larval MB. The larval MB consists of a single set of dorsal and medial lobes. Each of the MB neurons (highlighted in green) branches dendrites in the calyx and extends its axon through the peduncle, which bifurcates into the dorsal lobe (DL) and medial lobe (ML). (C) The olfactory network in the adult *Drosophila* brain. The antennal lobe (AL) is the first odor relay station for olfactory information in the fly brain, and consists of approximately 50 glomeruli that are identifiable as discrete neuropil groups. Dendrites of projection neurons (PNs) make specific connections in each different glomerulus in the AL. The PN axons convey olfactory information to higher brain centers by targeting the MB and the lateral horn (LH).

To analyze the functions of Unc-51 in neural development in the *Drosophila* brain, we first focused on the developing MBs at the larval stage (Fig. 1A). The cell bodies of the MBs (Kenyon cells) are localized to the dorsal cortex of the brain and extend dendrites into a structure called the calyx (Fig. 1B), which receives olfactory information from the projection neurons (PNs) of the antennal lobe (AL). The axons of the Kenyon cells elongate and fasciculate into a thick bundle to form the peduncle, a parallel axonal tract that extends ventrally and then splits into the dorsal and medial lobes in the larval brain (Fig. 1B). Internally, axons are organized in concentric layers in the developing MBs [28]. The temporal order of the generation of the Kenyon cells is reflected in the organization of the axons in the larval peduncle and lobes, in which the axons of newly born neurons first project into the center (the core) and then shift to the peripheral layers, as they mature [28].

### Unc-51 colocalizes with kinesin motor proteins in newly elongating MB axons

**Figure 2:**
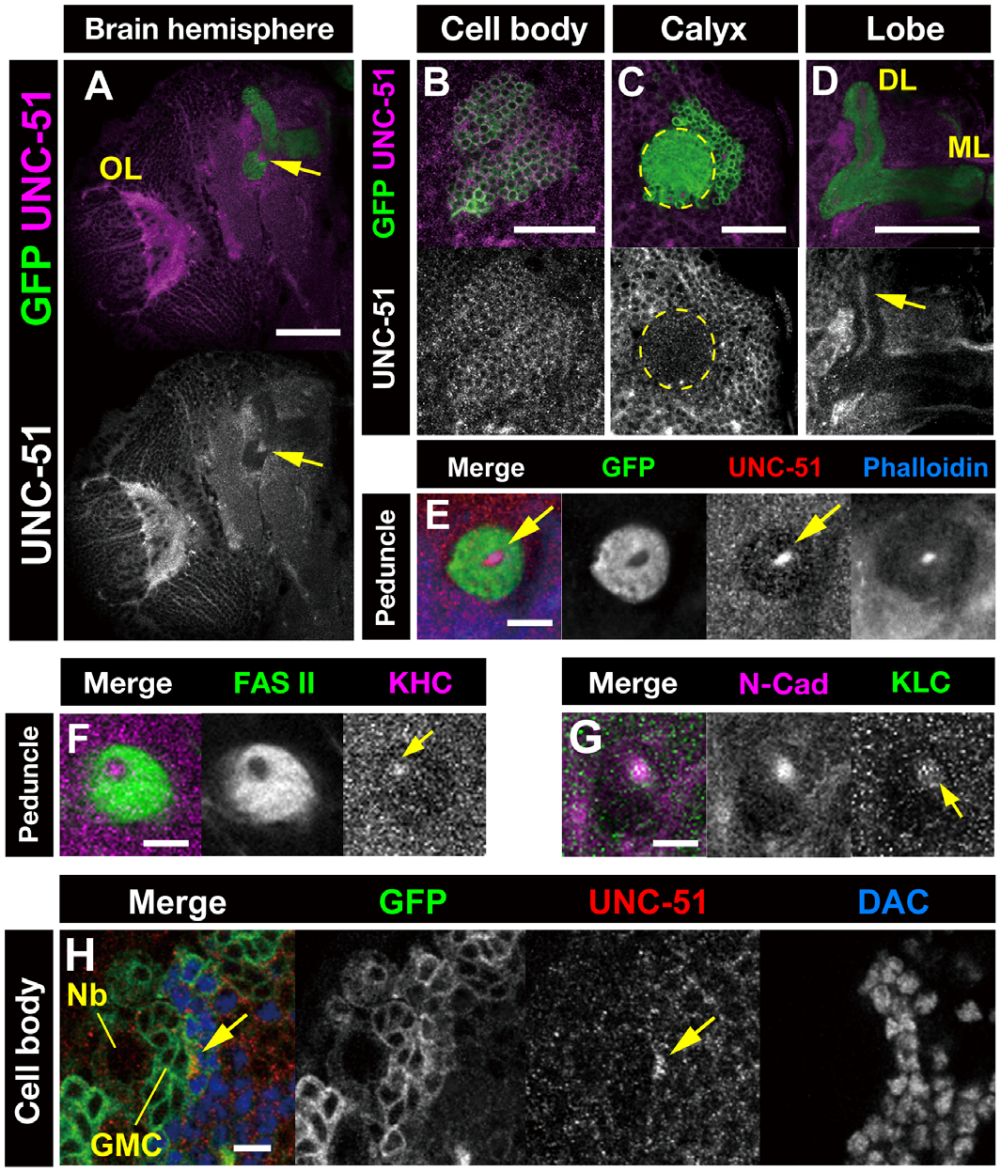
Expression of Unc-51 in the larval brain. (A–E) Late third instar larval brains stained with an anti-Unc-51 antibody (magenta). MB neurons are labeled with *UAS-mCD8::GFP* (green) driven by *OK107-GAL4*. (A) Brain hemisphere of a late third instar larva. OL, optic lobe. Arrows indicate the MB core. (B–D) Localization of Unc-51 in the MB cell bodies (B), calyx (C) and the lobe (D). Note that Unc-51 expression is detected in the cell bodies and the core fibers of the lobe (arrow in D) but not in the calyx (demarcated with a dashed circle). DL, dorsal lobe; ML, medial lobe. (E) Peduncle section showing the localization of Unc-51 in the core fibers, co-stained with phalloidin. *UAS-mCD8::GFP* (green) was driven by *OK107-GAL4*, which is expressed in outer layers. (F, G) Peduncle sections showing the localization of kinesin heavy chain (Khc) and kinesin light chain (Klc) in the core fibers (arrows). Note that Fas II is expressed in the outer layers while N-Cadherin (N-Cad) is expressed in the core fibers. (H) Higher magnification image of MB cell bodies. Arrow indicates elevated Unc-51 expression in newly differentiated cells located at the interface of the ganglion mother cells (GMCs) and the Dachshund (DAC)-positive postmitotic neurons. Nb, neuroblast. Cells were labeled with *UAS-mCD8::GFP* (green) driven by *elav-GAL4*. Scale bars: (A–D) 50 µm; (E–H) 10 µm.

To analyze the functions of Unc-51 in *Drosophila* brain development, we performed double/triple immunostaining of the larval brain with anti-Unc-51 antibody [16] and various neuronal markers. Although ubiquitous basal expression was detected in most part of the larval brain, elevated Unc-51 expression was detected in several regions, including the optic lobe (OL) and the MBs (Fig. 2A). In MBs, Unc-51 was enriched in the cell bodies (Fig. 2B), the core of the lobes and the peduncle (Fig. 2D, E). Sparse Unc-51 signals were also detected in the calyx and the outer layers of the peduncle. Double immunostaining with anti-Khc and anti-Fas II [28] anitibodies show that, as with Unc-51, Khc was preferentially expressed in the cor efibers (Fig. 2F). Similarly, double immunostaining with anti-Klc and anti-N-Cadherin antibodies [29] revealed that Klc also was preferentially expressed in the core fibers (Fig. 2G), suggesting active cytoplasmic transport by the kinesin motors in the newly elongating MB axons. Consistent with its expression in the core fibers, confocal optical sections of the MB cell bodies revealed that Unc-51 was enriched in the newly differentiated neurons that located at the interface of the ganglion mother cells and the postmitotic neurons that express Dachshund (Dac), a marker of differentiation (Fig. 2H).

### Loss of *unc-51* causes axonal transport defects in MB neurons

**Figure 3:**
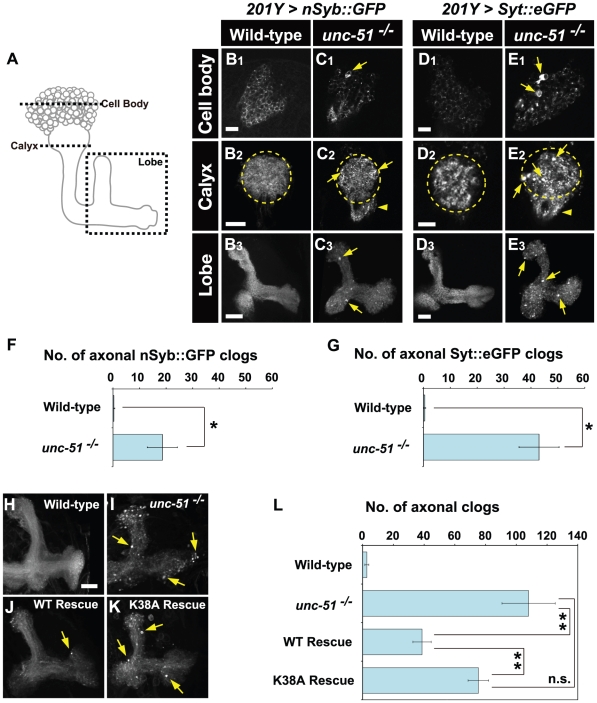
Loss of *unc-51* causes axonal transport defects in MB neurons. (A) Schematic representation of the larval MB. Dashed lines and box indicate the positions of the cross-sections presented in B–E. (B–E) Localization of n-Syb and Syt1 in the larval MB. Transport of n-Syb and Syt1 was monitored with green fluorescent protein (GFP) fusion constructs expressed in MB neurons using the *201Y-GAL4* driver. Note the accumulations of both markers in cell bodies (C1, E1; arrows), the calyx (C2, E2; arrows) and the lobes (C3, E3; arrows) in *unc-51* ^25/25^ mutant MBs. Dashed circles demarcate the calyx. Arrowheads in C2 and E2 indicate aberrant vesicle accumulation in the proximal peduncle. (F, G) Number of axonal clogs. Clog accumulations in the lobes were counted. (F) nSyb::GFP. (G) Syt::eGFP. (H–L) Unc-51 kinase activity is required for axonal transport in MB neurons. (H, I) mCD8::GFP distribution in wild type and *unc-51* ^25/25^ mutant MBs. Note the aberrant aggregates in the mutant lobes (arrows in I). (J, K) Genetic rescue of the axonal transport defect in *unc-51* mutant by an *unc-51* transgene. The axonal aggregation phenotype in *unc-51* ^25/25^ mutant was rescued by wild-type *unc-51* (J) but not by the kinase-deficient (K38A) mutant, *unc-51* ^K38A^ (K). *UAS-mCD8::GFP* and UAS-unc-51 were driven by *elav-GAL4*. Rescue genotypes: (J) *w; elav-GAL4*, *UAS-mCD8::GFP, UAS-unc-51* ^WT^; *unc-51* ^25/25^ and (K) *w; elav-GAL4, UAS-mCD8::GFP, UAS-unc-51* ^K38A^; *unc-51* ^25/25^. (L) The number of puncta observed in the lobe (mean ± SEM) was plotted for each genotype. **P<0.01 by Student's *t* test. n.s., not significant (*P* = 0.125). Scale bars, 10 µm.

In the segmental nerves of *Drosophila* larvae, Unc-51 plays a pivotal role in the regulation of axonal transport by mediating motor-cargo assembly [16]. Moreover, the observation that Unc-51 colocalizes with the kinesin motor proteins in the MB core fibers suggested that Unc-51 might have a regulatory function in axonal transport in MB neurons. To determine the functional importance of Unc-51 in MB neurons, we analyzed the intracellular transport of synaptic proteins in developing larval MBs. In wild-type MBs, both Synaptobrevin (n-Syb) and Syt 1 were detected in cell bodies, the calyx and the lobes, but not in the proximal peduncle (Fig. 3B, D). In contrast, aberrant accumulation of these synaptic vesicle proteins was detected in the cell bodies, the calyx and the proximal peduncle in most (10/12) *unc-51* mutant MBs (Fig. 3C, E; see clog quantification in F, G). None (0/8) of the wild-type MBs exhibited corresponding accumulations of the synaptic proteins. Toda et al. [16] have shown that *unc-51* mutations cause punctate accumulation of Khc in the segmental nerves. Consistent with this observation, loss of *unc-51* caused accumulation of Khc in the MB neurons (Fig. S1A, B; quantification in I, J). Mutations of *unc-51* also led to accumulations of Klc in the cell bodies, although punctate accumulation was less evident in the calyx and was rarely observed in the lobes (Fig. S1C, D; quantification in K and L). We also investigated the integrity of the microtubules in the *unc-51* mutant MBs by examining the expression and localization of a fluorescently labeled microtubule-associated protein marker, Tau::GFP [30]. No alterations were detected in the mutant MBs (*n* = 20) despite occasional accumulations in the cell bodies (Fig. S1E, F). Similarly, no accumulations were found in another microtubule-associated protein, Futsch [31], in the mutant MBs (Fig. S1G, H). These results suggest that, as in the segmental nerves, *unc-51* has an essential function in axonal transport in MB neurons.

Toda et al. [16] have shown that the kinase activity of Unc-51 is critical for its function in synaptic vesicle transport in segmental nerves. To determine whether Unc-51 kinase activity is similarly required in axonal transport in MB neurons, we performed genetic rescue experiments using the wild-type and a kinase-deficient form of the *unc-51* gene. *unc-51* mutant MBs displayed punctate accumulations of a fluorescently labeled membrane-bound marker, mCD8::GFP (Fig. 3H, I). The number of axonal accumulations in the mutant MBs was significantly suppressed by the expression of a wild-type *unc-51* (*unc-51* ^WT^) transgene (Fig. 3J) (*elav-GAL4*>UAS-*unc-51* ^WT^; *unc-51* ^25/25^) but not a kinase-deficient *unc-51* (*unc-51* ^K38A^) transgene (Fig. 3K) (*elav-GAL4*>UAS-*unc-51* ^K38A^; *unc-51* ^25/25^) (see Fig. 3L for quantification). These results confirm that the aberrant accumulation of membrane vesicles in developing MBs was indeed caused by the loss of Unc-51 activity, and suggest that the kinase activity of Unc-51 plays an important role in the phosphorylation-dependent regulation of axonal transport in MB neurons.

### Mutations in *unc-51* disrupt fasciculation of newly elongating axons in larval MBs

**Figure 4:**
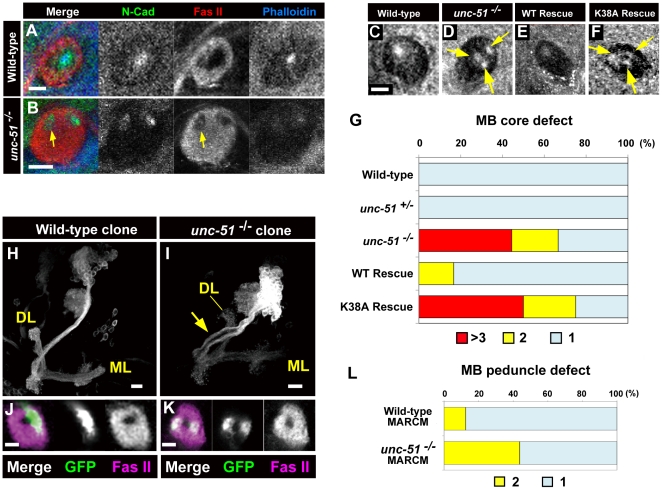
*unc-51* is required for fasciculation of MB axons. (A, B) Peduncle sections of late third instar MBs stained with anti-N-Cadherin (green), Fas II (red) and phalloidin (blue). (A) Wild type. (B) *unc-51* ^3/3^ mutant. Note the duplicate cores of the mutant. Whereas Fas II is expressed only in mature fibers surrounding the core in the wild type, in the mutant it is aberrantly expressed in a subset of the core fibers (arrow). (C–G) Peduncle sections showing that Unc-51 kinase activity is required for the fasciculation of core fibers. Core fibers were visualized with anti-N-Cadherin. (C) Wild type. (D) *unc-51* ^25/25^ mutant. The core defect was rescued by pan-neuronal expression of the *unc-51* ^WT^ transgene (E) but not by the kinase deficient (K38A) mutant (*unc-51* ^K38A^) (F). Genotypes: (E) *w; elav-GAL4*, *UAS-mCD8::GFP*, *UAS-unc-51* ^WT^; *unc-51* ^25/25^ and (F) *w; elav-GAL4*, *UAS-mCD8::GFP*, *UAS-unc-51* ^K38A^; *unc-51* ^25/25^. (G) Number of cores in the peduncle sections. Sixty-seven percent of the *unc-51* ^25/25^ mutant MBs had multiple core layers. The core defect was rescued by the *unc-51* ^WT^ but not the *unc-51* ^K38A^ transgene. Sample sizes: *n* = 16 for wild type, *n* = 18 for *unc-51* ^25/25^, *n* = 6 for *unc-51* ^WT^ rescue and *n* = 8 for *unc-51* ^K38A^ rescue. (H, I) Cell autonomous activity of *unc-51* is required for the fasciculation of MB axons. Wild type (H) and *unc-51* ^3/3^ mutant (I) neuroblast clones. Mutant clones have wild type-like dorsal lobe (DL) and medial lobe (ML), but exhibit de-fasciculation of the peduncular axons (arrow). (J, K) Cross-sections of the wild-type (J) and mutant (K) peduncles. Clones were induced by a heat shock at the early first instar stage and labeled with mCD8::GFP (green) driven by *elav* ^c155^. Mature axons were stained with anti-Fas II (magenta). (L) Quantification of the number of peduncular axons in MARCM clones. While only 13% of the wild-type clones (*n* = 8) exhibited separate bundles, 44% of the *unc-51^−/−^* clones (*n* = 16) demonstrated split fascicles. Scale bars, 10 µm.

The characteristic expression of Unc-51 in the newly differentiated MB axons suggested that Unc-51 might have a pivotal function in the integrity of the MB core. To investigate the role of Unc-51 in MB development, we analyzed the anatomical phenotypes of *unc-51* mutants using several axonal markers. Immunological examinations with phalloidin or anti-N-Cadherin [29] staining showed that the wild-type larval MBs always had a single core (Fig. 4A). In contrast, the cores of *unc-51* mutant MBs had disintegrated into multiple bundles, with 60% of the mutant MBs showing more than two cores (Fig. 4B and quantification in G). Moreover, aberrant expression of Fas II was often detected in core axons (arrow in Fig. 4B). Of note, the defective cores in the *unc-51* mutant MBs were rescued by the *unc-51* ^WT^ transgene but not by the kinase-deficient *unc-51* ^K38A^ transgene (Fig. 4C–F and quantification in G). These results reveal the critical role of Unc-51 kinase activity in the fasciculation of growing axons in larval MBs.

Because Unc-51 expression was not specific to MB neurons in the larval brain (Fig. 2A), we then asked whether the activity of *unc-51* was required cell autonomously, by generating *unc-51*-null clones in an otherwise heterozygous genetic background. Wild-type and mutant clones were induced using the Mosaic Analysis with a Repressive Marker (MARCM) method [32], and labeled with *UAS-mCD8::GFP*. Whereas the majority of the wild-type clones (7/8) maintained a single peduncular fascicle (Fig. 4H, J; quantification in L), 44% of the mutant clones (7/16) exhibited split fascicles (Fig. 4I, K; quantification in L). On the other hand, both the dorsal and the medial lobes were formed in the mutant clones as in the wild-type clones. This suggests that cell-autonomous activity of *unc-51* is indeed critical in maintaining fasciculation of growing axons in larval MBs.

### Mutations in *unc-51* cause dendritic targeting defects in MBs

**Figure 5:**
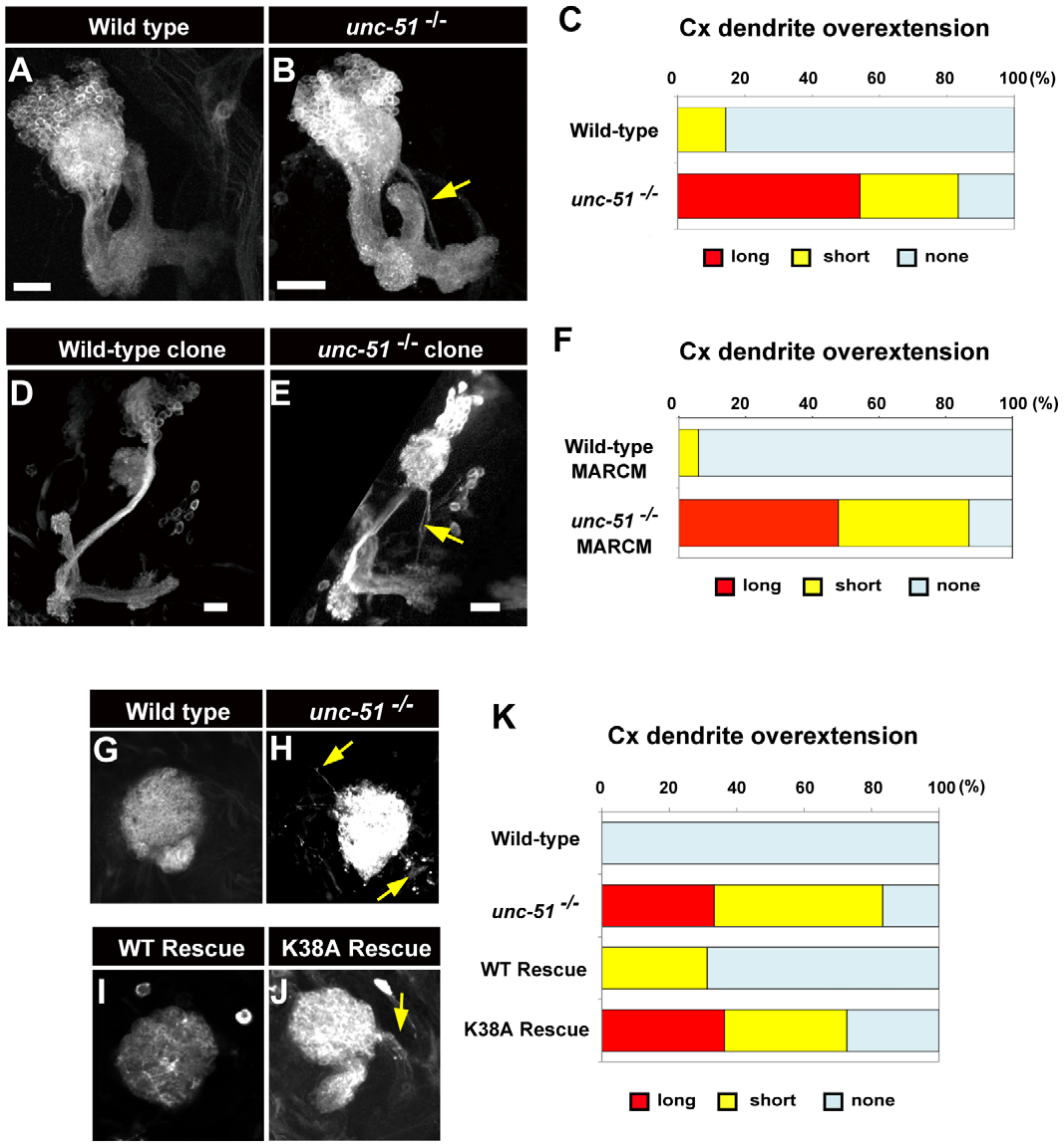
Loss of *unc-51* causes dendritic defects in MBs. (A, B) MBs in the wild-type and *unc-51* ^25/25^ mutant larvae at the late third instar stage. MBs were visualized with mCD8::GFP driven by 201Y. (C) Quantification of the dendrite overextension phenotype in wild-type and *unc-51* mutant larvae. While 14% of the wild-type MBs (*n* = 21) exhibited short overextensions, 83% of the *unc-51* ^−/−^ MBs (*n* = 24) exhibited short or long overextensions (shorter or longer than the diameter of the calyx, respectively). (D, E) Analyses of the dendritic phenotype using MARCM clones. Clones were induced by a heat shock at the early first instar stage. MB neurons were labeled with *UAS-mCD8::GFP* driven by *elav* ^c155^. (D) Wild-type neuroblast clone. (E) *unc-51* ^3/3^ mutant neuroblast clone. (F) Quantification of the dendrite overextension phenotype in MARCM clones. While only 6% of the wild-type clones (*n* = 17) showed overextensions, all of the *unc-51* ^3/3^ clones (*n* = 23) exhibited short or long overextensions (shorter or longer than the diameter of the calyx, respectively). (G–J) Dendrite overextension phenotype in *unc-51* mutant MBs was rescued by pan-neuronal expression of *unc-51* ^WT^ but not by the kinase-deficient (K38A) *unc-51* ^K38A^ transgene. (G) Wild-type. (H) *unc-51* ^25/25^ mutant. Rescue genotypes: (I) *w; elav-GAL4*, *UAS-mCD8::GFP*, *UAS-unc-51* ^WT^; *unc-51* ^25/25^ and (J) *w; elav-GAL4*, *UAS-mCD8::GFP*, *UAS-unc-51* ^K38A^; *unc-51* ^25/25^. (K) Quantification of the dendrite overextension phenotype. While none of the wild-type MBs (*elav-GAL4*, *UAS-mCD8::GFP*) (*n* = 6) exhibited calyx overextension, 83% of the *unc-51* ^25/25^ mutant MBs (*elav-GAL4*, *UAS-mCD8::GFP*; *unc-51* ^25/25^) exhibited short or long overextensions (*n* = 6). The dendritic overextension phenotype was significantly suppressed by the *unc-51* ^WT^ transgene (*w; elav-GAL4*, *UAS-mCD8::GFP*, *UAS-unc-51* ^WT^; *unc-51* ^25/25^), with 31% of the MBs exhibiting short extensions (*n* = 16). Expression of *unc-51* ^K38A^ failed to rescue the phenotype (*w; elav-GAL4*, *UAS-mCD8::GFP*, *UAS-unc-51* ^K38A^; *unc-51* ^25/25^), with 73% of the MBs exhibiting short or long overextensions (*n* = 22). Note different genetic background from that of the experiments in (A–C). Arrows in (B, E, H and J) indicate overextensions from the calyx. Scale bar, 10 µm.

In addition to the fasciculation defects, mutations in *unc-51* also caused aberrant projections from the calyx. Approximately 83% of the *unc-51* mutant MBs (*n* = 24) exhibited overextension of calyx fibers. Among them, more than half (54%) of the MBs exhibited overextensions longer than the calyx diameter. On the other hand, the wild-type MBs rarely exhibited calyx overextensions, with only short extensions (3/21; shorter than the calyx diameter) (Fig. 5A–C). Analyses with wild-type and mutant MARCM clones suggested that cell-autonomous activity of *unc-51* was required for the suppression of dendritic overshooting (Fig. 5D, E); most of the *unc-51* mutant clones exhibited either long (48%) or short (39%) overextensions, while wild-type clones exhibiting short extensions were very rare (6%) (Fig. 5F).

The kinase activity of Unc-51 was again critical to suppress dendritic overextension from the calyx (Fig. 5G–J). Expression of the *unc-51* ^WT^ transgene markedly suppressed the calyx overextension defects while expression of the kinase-deficient *unc-51* ^K38A^ transgene failed to rescue the phenotype (Fig. 5G–K). This implies an additional role for Unc-51 kinase activity in confining dendritic extensions of MB neurons within the normal dendritic compartment.

### Mutations in *unc-51* cause axonal and dendritic targeting defects in AL-PNs

To further investigate the importance of *unc-51* in axonal and dendritic development, we analyzed the development of the AL-PNs in the adult brain, which convey olfactory information to higher brain centers by targeting stereotypic locations in the MBs and the lateral horn (LH) (Fig. 1C) [33]. Using a *GH146-GAL4* driver, which is expressed in approximately 90 of the estimated 150 PNs [34], we generated PN-MARCM clones that were homozygous for *unc-51* mutation.

The wild-type AL-PN axons were bundled in a single fascicle, extending from the AL to the LH (Fig. S2A, C). Although most of the *unc-51^−/−^* anterodorsal neuroblast clones (0/11) failed to show clear defects in axonal projections (Fig. S2B), 71% of the *unc-51* lateral neuroblast clones (5/7) exhibited fasciculation defects (Fig. S2D). We also generated wild-type and mutant single-cell clones that innervated the DL1 glomerulus (Fig. S2E, F). Analyses with mutant DL1 single-cell clones showed that the number of branches in the calyx was slightly increased in the mutant clones (wild type = 6.4; *unc-51* mutant = 7.8, *p*<0.05) while the axonal projection patterns were mostly unaffected, with a normal number of terminal branches in the LH (Fig. S2G, H).

Reminiscent of the dendritic phenotype in the mutant MB calyces, many of the *unc-51^−/−^* mutant PNs (12/22) exhibited overshooting of their dendrite terminals beyond the AL compartment, while none of the wild-type clones (0/14) exhibited the corresponding phenotype (Fig. S3). Overshooting was observed for all three types of neuroblast clone (anterodorsal, lateral, and ventral neuroblasts) (Fig. S3B, D, F) with some of the mutant PN clones having multiple extensions (Fig. S3D).

To investigate the regulatory functions of *unc-51* in dendritic development further, we analyzed the precise targeting phenotypes of the mutant PN clones to the individual glomeruli. We focused on 12 landmark glomeruli that are innervated by GH146-positive anterodorsal PNs and are clearly identifiable by their positions and shapes [35]. Eight of the landmark glomeruli (VA1d, VA1lm, VA3, VM2, DM6, D, DL1, VM7) were normally innervated by the wild-type anterodorsal PNs, whereas four (VA2, DA1, DM5, DM2) were never or rarely innervated (Fig. S4A, C, E, K). Although *unc-51* anterodorsal neuroblast clones exhibited complete innervation of all the on-target glomeruli (Fig. S4B, D, F, M), they often showed mild to severe ectopic innervation of the off-target glomeruli DA1, DM5 and DM2 (Fig. S4B, F, N). These results suggest that *unc-51* is dispensable for correct targeting of on-target glomeruli but is required for restriction of the dendritic arbors within the normal target glomeruli, by suppressing ectopic dendritic innervation.

### *unc-51* mutant MB neurons exhibit aberrant accumulation of endosomes and Golgi

To investigate the mechanisms underlying dendritic abnormalities, we analyzed the localization of endosomes and the Golgi. The small GTPase Rab5, a component of early endosomes, plays a pivotal role in dendritic branching of the larval peripheral sensory neurons [10]. Moreover, Unc51.1 regulates Rab5-mediated endocytic pathways in developing granule cells in mice [14]. In the wild-type larval brain, punctate signals of Rab5-containing endosomes were detected in the cell bodies and the calyx of the MBs (Fig. S5A). In contrast, increased numbers of Rab5-containing puncta and irregular accumulations were observed in the cell bodies and the calyx of the mutant MBs. Punctate Rab5 signals were also detected in the mutant MB lobes, while only homogeneous staining was detected in the wild-type MB lobes (Fig. S5B, G). Similarly, loss of *unc-51* caused aberrant accumulations of Lamp1, a late endosome marker, throughout the MBs (Fig. S5D, H). The Golgi secretory pathway plays a critical role in dendritic growth and branching [5], [7], [8]. We examined localization of the Golgi machinery using a fluorescently labeled Golgi marker, α-Man-II::eGFP [7]. In the wild-type MB neurons, punctate α-Man-II::eGFP signals were detected in the cell bodies but not in the calyx and the lobes (Fig. S5E). On the other hand, ectopic punctate signals and accumulations of the Golgi marker were detected in the calyx and the lobes of the *unc-51* mutant MB neurons (Fig. S5F, I). Aberrant accumulations were also detected in the cell bodies. These results suggest that *unc-51* has an essential function in intracellular trafficking and localization of endosomes and Golgi components in developing MB neurons.

In contrast to the significant transport defects of endosomes and Golgi components, accumulation of Mito::GFP, a fluorescently labeled mitochondrial marker [36], was rarely observed in the mutant MBs, although ectopic signals were detected in the proximal peduncle (Fig. S6A, B). We also examined the expression of hLC3::GFP, a fluorescently labeled marker of autophagosomes [37], [38], [39], and observed no difference between the wild-type and mutant MBs (Fig. S6C, D).

### Loss of *unc-51* causes mislocalization of Fas II in MB neurons

**Figure 6:**
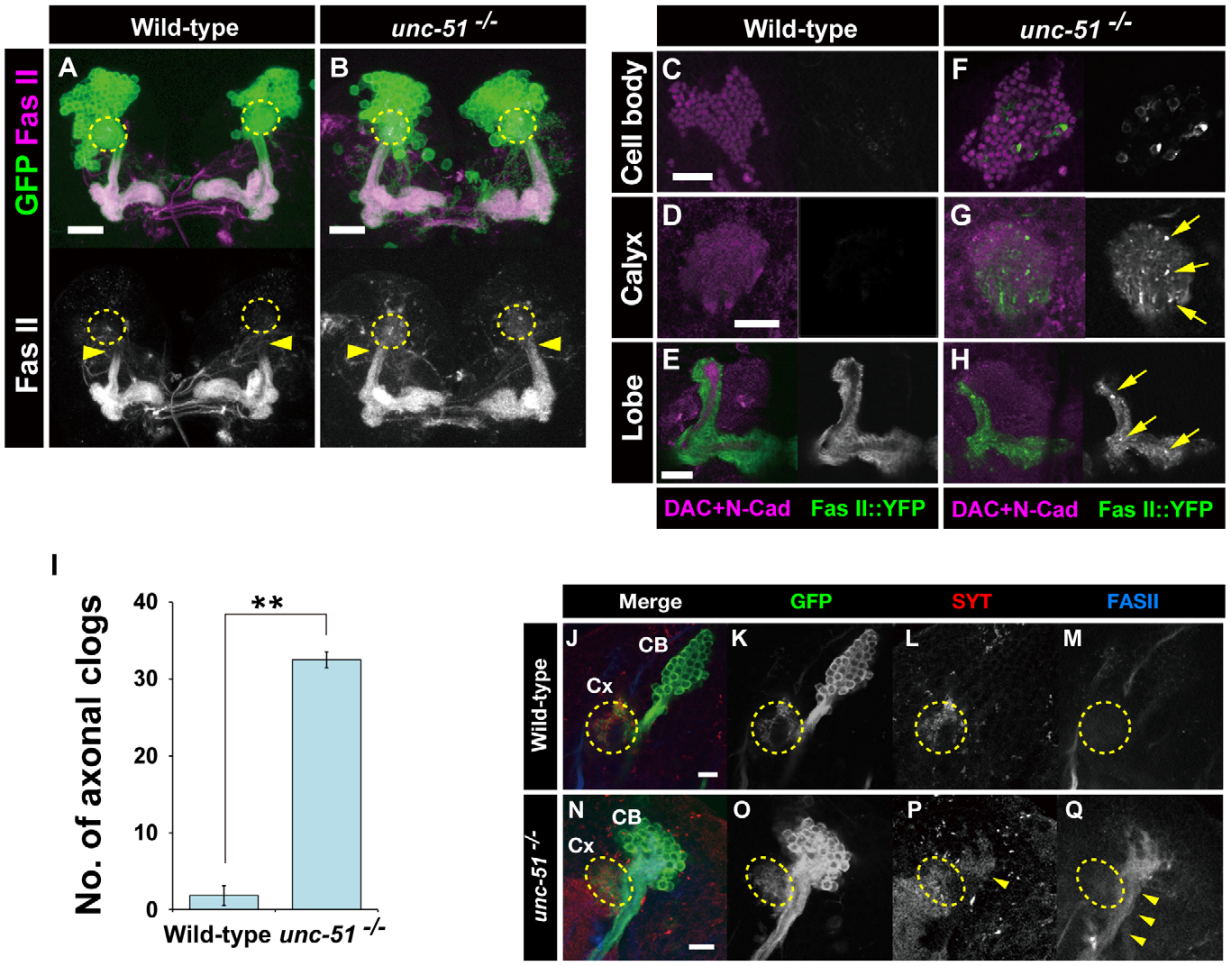
*unc-51* regulates the subcellular localization of Fas II. (A, B) Localization of Fas II in the wild-type and *unc-51* ^25/25^ MBs. In wild type, Fas II (magenta) was localized only to the lobes and the distal peduncle. In *unc-51* ^25/25^ mutant MBs, Fas II was mislocalized to the calyx (yellow dashed circles) and the proximal peduncles (arrowheads). MBs were labeled with *UAS-mCD8::GFP* (green) driven by *201Y-GAL4*. (C–H) Localization of Fas II monitored with a YFP fusion construct. Fas II::YFP transgene (green, white) was expressed by the *201Y-GAL4* driver. Counterstained with anti-Dac and anti-N-Cadherin antibodies to visualize the MB neurons (magenta). In wild type (C–E), Fas II::YFP was localized to the lobes and the distal peduncle (not shown), as was the endogenous protein. Loss of *unc-51* caused mislocalization of Fas II in the calyx (G). Aberrant Fas II::YFP accumulations (arrows) were observed in the cell bodies (F) and the lobes (H). Note that the internal core was disrupted in the mutant lobes (H). (I) Quantification of the number of axonal clogs in the lobes of the wild type and *unc-51^−/−^* mutant clones. (J–Q) Cell autonomous activity of *unc-51* is required for intracellular transport of Fas II and Syt1. Wild-type (J–M) and *unc-51* ^3/3^ mutant (N–Q) neuroblast clones. Clones were induced by an early first instar heat shock and labeled with *UAS-mCD8::GFP* driven by *elav* ^c155^ (green). Fas II was mislocalized in the proximal part of the axons in all the mutant clones (13/13) (arrowheads in M). Most of the mutant clones also exhibited ectopic Syt1 accumulation (10/12) in the proximal part of the MB axons (arrowhead in P). None of the wild-type clones accumulated Fas II (0/9) or Syt1 (0/8) in the corresponding regions. CB, cell bodies; Cx, calyx (indicated by yellow dashed circles). Scale bar, 10 µm.

Fas II plays a critical role in the development of the concentric layers in the MBs [28]. In the wild-type larval MBs, Fas II was localized to the lobes and the distal peduncles but absent from the calyx and the proximal peduncle (arrowheads in Fig. 6A). Although Fas II localization to the distal peduncle was not altered, mutations in *unc-51* caused mislocalization of Fas II to the proximal peduncle (arrowheads in Fig. 6B). Moreover, Fas II was detected in the calyx of *unc-51^−/−^* mutant MBs (Fig. 6B). To further investigate the localization of Fas II in the developing MBs, we examined Fas II distribution using a Fas II::yellow fluorescent protein (YFP) fusion construct driven by the MB-GAL4 driver, 201Y. In the wild-type MBs, Fas II::YFP was localized to the lobes as was the endogenous protein, but was absent from the cell bodies and the calyx (Fig. 6C–E). In contrast, Fas II::YFP aberrantly accumulated in the cell bodies and the calyx in the *unc-51* mutant MBs (Fig. 6F, G). In addition, punctate accumulations of Fas II::YFP were detected in the mutant calyx and the lobes (Fig. 6H; quantification in I). Furthermore, clonal studies demonstrated that the *unc-51^−/−^* mutant clones (13/13) exhibited aberrant localization of Fas II in the calyx and the proximal peduncle (Fig. 6N–Q) whereas no wild-type clone (0/9) showed corresponding mislocalization (Fig. 6J–M). This demonstrates that cell-autonomous activity of *unc-51* is required for correct localization of Fas II in the larval MB neurons.

### Suppression of kinesin-mediated transport results in dendritic targeting defects in MB neurons

While the above results suggest that *unc-51* regulates intracellular trafficking of various vesicles and molecular cargos in developing MB neurons, previous studies have suggested multiple roles for *unc-51* in diverse cellular and neuronal processes [12], [13], [15], [17], [23], [37], [38], [40], [41], [42], [43]. To examine whether kinesin-mediated transport plays a critical role in developing MB neurons, we studied *Klc* and *unc-51* double heterozygous flies, based on the fact that *unc-51* genetically interacts with *Klc* in kinesin-mediated axonal transport in the segmental nerves [16].

**Figure 7:**
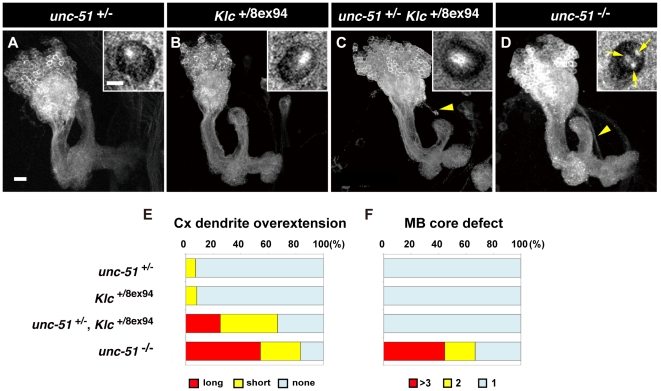
*unc-51* genetically interacts with *Klc* in dendrite development. (A–D) Larval MBs of single or double heterozygous mutants. Late third instar stage. MBs were labeled with *UAS-mCD8::GFP* driven by *201Y-GAL4*. Inset panel shows a peduncle cross-section stained with anti-N-Cadherin. Arrowheads indicate overextending dendrites. Arrows in the inset of (D) indicate multiple MB cores in a *unc-51* ^25/25^ larva. (E) Quantification of dendritic targeting defects. Sample sizes: *unc-51* ^+/25^ (*n* = 15), *Klc^+/^* ^8e×94^ (*n* = 27), *Klc^+/^* ^8e×94^ *unc-51* ^+/25^ (*n* = 53) and *unc-51* ^25/25^ (*n* = 21). (F) Quantification of MB core defects. Sample sizes: *unc-51* ^+/25^ (*n* = 13), *Klc^+/^* ^8e×94^ (*n* = 8), *unc-51* ^+/25^ *Klc* ^+/8e×94^ (*n* = 12) and *unc-51* ^25/25^ (*n* = 18). Scale bars, 10 µm.

**Figure 8:**
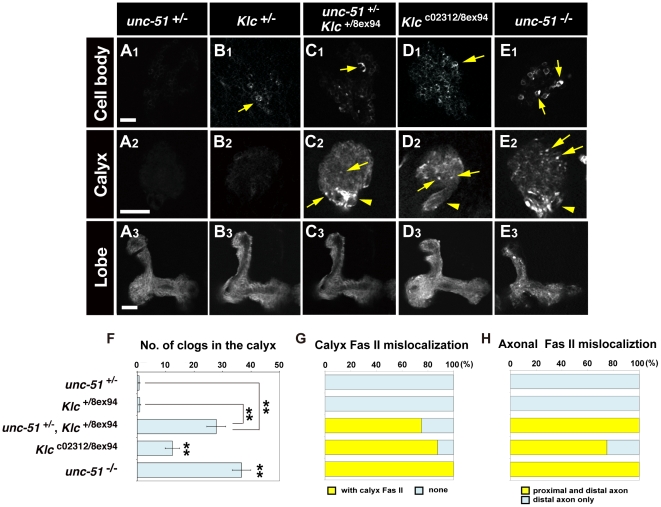
Aberrant Fas II accumulation and mislocalization in *unc-51* and *Klc* mutants. (A–E) Fas II::YFP distribution patterns in the cell bodies (A1–E1), calyx (A2–E2) and lobes (A3–E3). Fas II::YFP was expressed in MBs using the *201Y-GAL4* driver. Yellow arrows indicate aberrant Fas II clogs. Arrowheads (C2, D2 and E2) indicate Fas II mislocalization in the proximal peduncle. Scale bars, 10 µm. (F) Number of clogs in the calyx. Error bars, mean ± SEM. **P<0.01, Student's *t* test. Comparisons were also made between *Klc* ^c02312/8e×94^ and *Klc* ^+/8e×94^ (**P<0.01) as well as between *unc-51* ^25/25^ and *unc-51* ^+/25^ (**P<0.01). (G) Quantification of Fas II::YFP mislocalization in the calyx. (H) Quantification of Fas II mislocalization in the proximal peduncle. Sample sizes: *unc-51* ^+/25^ (*n* = 6), *Klc* ^+/8e×94^ (*n* = 14), *unc-51* ^+/25^ *Klc* ^+/8e×94^ (*n* = 4), *Klc* ^c02312/8e×94^ (*n* = 8) and *unc-51* ^25/25^ (*n* = 8).

While calyx abnormality was rarely observed in the MBs of either *unc-51* ^+/−^ (7%, *n* = 15) or *Klc* ^+/−^ (8%, *n* = 27) single heterozygotes (Fig. 7A, B), many of the MBs of *Klc* ^+/−^ *unc-51* ^+/−^ double heterozygotes exhibited overshooting (56%, *n* = 53) (Fig. 7C; quantification in E), reminiscent of the MB phenotypes in *unc-51* ^−/−^ homozygotes. On the other hand, axonal fasciculation in the MB core was barely affected in the *Klc* ^+/−^ *unc-51* ^+/−^ double heterozygotes (inset in Fig. 7C; quantification in F). As in the MBs of *unc-51* ^−/−^ homozygotes, Fas II localization was also altered in the MBs of *Klc* ^+/−^ *unc-51* ^+/−^ double heterozygotes. Accumulation of Fas II was rarely detected in either the *Klc* ^+/−^ or *unc-51* ^+/−^ single heterozygotes (Fig. 8A, B), but the majority of *Klc* ^+/−^ *unc-51* ^+/−^ double heterozygotes exhibited aberrant accumulations and mislocalization of Fas II in the MBs (Fig. 8C), as did the *unc-51* homozygotes (Fig. 8E; quantification in F–H).

To further investigate functional importance of the kinesin motors in MB development, we examined MBs of *Klc* mutant larvae. Because of the homozygous lethality of the *Klc* ^8e×94^ null allele [44], we used trans-heterozygotes with *Klc* ^c02312^, a hypomorphic P-element insertion allele [45]. Notably, *Klc* ^8e×94^/*Klc* ^c02312^ larvae exhibited weak but significant Fas II accumulations in the MBs (Fig. 8D and F), suggesting that kinesin-mediated intracellular transport plays an essential role in MB development. In addition, as with the *unc-51* ^−/−^ larvae, *Klc* ^8e×94^/*Klc* ^c02312^ larvae showed mislocalization of Fas II in the calyx and the proximal peduncle (Fig. 8D2; quantification in G, H). Moreover, the internal core structure was partially disrupted in the *Klc* ^8e×94^/*Klc* ^c02312^ trans-heterozygotes (Fig. 8D3).

To further confirm the functional requirement of kinesin-mediated transport, we generated *Klc* ^8e×94^ MARCM clones, and found that loss of the *Klc* activity caused disintegration of the axonal bundles and aberrant dendritic overshooting (Fig. S7A–F). *Klc* ^8e×94^ clones also exhibited mislocalization of Fas II in the proximal part of the peduncle, recapturing the phenotype of the *unc-51^−/−^* clones (Fig. S7G). These results thus clearly demonstrate that cell-autonomous activity of the kinesin motor protein is critical for the normal axodendritic development of MB neurons.

Kurusu et al. [46] have shown that members of the receptor tyrosine phosphatase (RPTP) protein family play a critical role in axonal fasciculation and elongation of the core fibers in developing MBs. To examine possible interaction of *unc-51* and the *RPTP* genes, we performed genetic interaction experiments between the *unc-51* and the *RPTP* mutations but failed to detect any abnormalities in the MBs of double heterozygotes for *unc-51* and either *Ptp10D*, *Ptp69D* or *Lar*. Having a wild-type like lobes with a single distinctive core, none of the heterozygotes exhibited abnormality in the fasciculation of MB axons (Fig. S8). Neither aberrant Fas II accumulation nor dendritic abnormality was caused in these double heterozygote larvae.

### *unc-51* is dispensable for polarized transport of Dscam and Nod

Having demonstrated the importance of *unc-51* in the kinesin-mediated intracellular transport in the development of MB neurons, we then asked whether *unc-51* was required for the localization of proteins that are distinctively localized to dendrites by retrograde transport motors. Among the multiple isoforms of the Dscam proteins, isoforms carrying exon 17.1 (Dscam[TM1]), which encodes a transmembrane domain, are targeted to dendrites by dynein/dynactin-mediated transport [47], [48], [49]. In wild-type MBs, Dscam[TM1] was localized specifically to the calyx and absent from the lobes (Fig. S9A–C). In the *unc-51* mutant MBs, Dscam[TM1] partly accumulated in cell bodies but nonetheless was normally targeted to the dendrites, with no detectable signals in the lobes in all the samples examined (*n* = 12) (Fig. S9D–F; see clog quantification in M, N).

To examine retrograde dendritic transport further, we analyzed the localization of Nod::β-Gal, a chimera comprising β-galactosidase fused to the Nod motor domain, which binds to microtubule minus ends [50]. In wild-type MBs, Nod::β-Gal was detected in cell bodies and specifically targeted to the calyx with virtually no signals in the lobes (Fig. S9G–I), confirming previous observations [9], [51], [52]. Although *unc-51* mutant MBs exhibited accumulations in the cell bodies, Nod::β-Gal was specifically targeted to the calyx in all the samples examined (*n* = 14) (Fig. S9J–L; see clog quantification in O, P). Of note, Nod::β-Gal was observed in the tips of the overextensions of the *unc-51* mutant calyx (arrowheads in Fig. S9K), arguing for their minus end polarity and thus dendritic identity. These results imply that the activity of *unc-51* is dispensable for polarized dynein/dynactin-mediated retrograde transport in dendrites. They also argue that the microtubule system in mutant MB neurons is functionally intact.

## Discussion

Active transport and delivery of molecular and cellular components from the soma to the distinct cytoplasmic compartments is critical not only for synaptic function in mature neurons but also for axonal elongation and guidance in developing neurons [1], [3], [6]. The kinesin-mediated anterograde transport plays a major role in the active traffic in the developing neurons, delivering a wide range of cargos along the axon, including synaptic vesicles, mitochondria, cytoskeletal elements, and mRNAs.

### Unc-51 controls membrane vesicle transport in developing brain *via* kinesin-mediated transport

In this work, we have demonstrated preferential expression of Unc-51 and kinesin motor proteins in larval MBs, and shown that loss of *unc-51* activity causes severe defects in kinesin-mediated transport in developing MB neurons, while dynein/dynactin-mediated retrograde transport is unaffected in *unc-51* mutant MBs. In addition, loss of *unc-51* activity results in disintegration of axonal bundles and aberrant extensions of dendrites in both MB and AL neurons. These results suggest that *unc-51* is essential for the development of brain neurocircuitries, participating in molecular and/or cellular mechanisms that regulate the formation of the complex structures such as MBs. Indeed, our results demonstrate that *unc-51* is essential for the specific intracellular localization of axonal fasciculation and guidance molecules such as Fas II.

Although previous studies have demonstrated that *unc-51* has diverse functions in developing neurons [12], [13], [15], [17], [23], [37], [38], [40], [41], [42], [43], our analyses of the *Klc* ^+/−^ *unc-51* ^+/−^ double heterozygotes clearly demonstrate that suppression of kinesin-mediated transport results in dendritic overextensions. Concomitant suppression of *unc-51* and *Klc* also causes axonal transport defects that are reminiscent of *unc-51* mutants. Furthermore, the double heterozygotes exhibit mislocalization of Fas II in both the calyx and the proximal peduncle. This recapitulates the salient phenotype of *unc-51* ^−/−^ mutants, and argues that defective kinesin-mediated transport is the major molecular process that underlies the developmental defects in the *unc-51* mutants. On the other hand, *Klc* ^+/−^ *unc-51* ^+/−^ double heterozygotes failed to exhibit a full range of the *unc-51* mutant phenotypes. Although we cannot exclude the possibility that other molecular processes are involved in the *unc-51* mutant phenotypes, the weaker phenotypes of the double heterozygotes might be accounted by partial suppression of the gene activity given that wild-type alleles are retained at a half dosage for both genes. Furthermore, the result that *Klc* null mutant clones exhibited dendritic and axonal defects that were reminiscent of the *unc-51* mutant clones clearly confirms the importance of the kinesin-mediated transport in brain development. It is also noteworthy that, as with *Klc* mutant clones, *Khc* null MB clones exhibit similar yet more pronounced defects, although the critical requirement of the *Khc* activity for neuroblast division hampers a precise assessment of *Khc* function in axodendritic development (H. M. and K. F. T., unpublished observation). These results as a whole highlight the importance of kinesin-mediated vesicle transport in the development and wiring of complex networks in the brain.

Previously, Toda et al. [16] demonstrated that *unc-51* plays an essential role in axonal transport by mediating the assembly of the cargos and the kinesin motor proteins. In *unc-51* mutants, membrane vesicle transport is severely affected, resulting in accumulation of synaptic vesicles in the larval segmental nerves. Loss of *unc-51* activity also causes aggregation of Rab5-positive membranes in the segmental nerves [16]. Notably, as in the mutant MBs, kinesin motors are accumulated in the mutant segmental nerves while overall mitochondrial localization was unaffected [16]. Genetic studies have shown that the wild-type but not a kinase-deficient form of the *unc-51* transgene rescues the transport defect in synaptic vesicles in the mutant segmental nerves [16]. Moreover, the kinase activity of Unc-51 is critical for phosphorylation of an adaptor molecule, Unc-76/FEZ, which mediates the assembly between synaptic vesicle cargos and the kinesin motor complex. Our finding that the dendritic and axonal defects in MB neurons are rescued by the wild-type but not by the kinase-deficient *unc-51* transgene suggests that a similar phosphorylation-dependent regulatory mechanism is involved in the intracellular transport in developing MB neurons. In line with this, it is noteworthy that Unc-76 is preferentially expressed in developing MB neurons, in which it colocalizes with Unc-51 and kinesin motor proteins in the core fibers (H. M. and K. F. T., unpublished observation). Intriguingly, both Unc-51 and Unc-76 are downregulated in the mature neurons that surround the core fibers as they mature and shift to the peripheral layers. Concomitantly, both Khc and Klc are downregulated in the mature MB neurons, suggesting dynamic control of the expression of the molecular components that mediate active vesicle transport in developing MB neurons.

### Kinesin-mediated vesicle transport in dendritic development

Recent genetic studies on the dendritic development of *Drosophila* larval sensory neurons showed that the microtubule motor protein dynein controls dendritic branching by directing polarized intracellular vesicle trafficking [9], [10]. Dynein is also necessary for the dendrite-specific localization of Golgi outposts [9], which secretory pathway plays critical roles in dendritic growth and branching [5], [7], [8]. These studies also showed that Rab5 was essential to normal dendritic branching, *via* its role in controlling endosomal trafficking [10]. Our results show that mutations of *unc-51* leads to aberrant accumulations of Rab5-containing endosomes and Golgi components in developing larval MBs. On the other hand, polarized dendritic transport of Dscam is not altered in the *unc-51* mutant MBs, implying that retrograde dynein/dynactin-mediated transport remains intact in the mutant MB neurons. Moreover, another dendritic molecule, Nod, is correctly targeted to the calyx, clearly indicating that *unc-51* is not required for polarized retrograde transport mediated by dynein/dynactin in developing MB neurons. These results are consistent with our previous observations that *unc-51* fails to interact with retrograde motor genes such as *dynein heavy chain* and *Lissencephaly-1* in segmental nerves [16].

On the other hand, Satoh et al. [10] found that *khc* mutants showed dendritic branch abnormalities that were almost identical to those of *dynein light intermediate chain (Dlic)* mutants, with reduced arbors and a marked shift in branching activity in the proximal area within the arbors. In the *khc* mutants, the dynein complexes become aggregated distally, suggesting that kinesin plays a role in recycling dynein proximally after it has carried its organelle cargo distally. These phenotypes seem to contrast with those observed in *unc-51* mutant MB neurons, which show dendritic overextensions with normal dynein/dynactin-mediated transport. The exact mechanisms underlying these discrepancies are unknown, but this might suggest different regulatory processes for kinesin-mediated transport that operate in the distinct cellular contexts of the peripheral sensory neurons and MB neurons. Toda et al. [16] showed that *unc-51* mutation resulted in varying degrees of axonal membrane defects that were dependent on the cargo. It is possible that Unc-51 differentially regulates the transport of specific cargo subsets by phosphorylation of distinct groups of adaptor proteins in different cell types.

### Unc-51 controls axonal growth and guidance by endosomal vesicle trafficking

Studies in *Drosophila* identified *unc-51* as a homolog of the yeast *atg1*, and suggested that Unc-51 kinase activity is required for the induction of autophagy [37], [38], [39]. Recently, Shen and Ganetzky [43] showed that autophagy positively regulates synapse development at the *Drosophila* neuromuscular junction. Mutations in autophagy genes including *atg1/unc-51*, caused significant reduction in terminal branching of the segmental motoneurons, with reduced numbers of boutons. In contrast, our single-cell analysis of AL-PNs (Fig. S2) shows that loss of *unc-51* activity results in an increase in the number of the calyx branches. Our results also demonstrate that the distribution of an autophagy marker is not altered between the wild-type and the *unc-51* mutant MBs (Fig. S6). These results argue against autophagy as an underlying mechanism of the axodendritic abnormalities in the *unc-51* mutant larval brain, and are consistent with a previous report that autophagy is not involved in Unc-51-mediated regulation of axonal transport [16].

In *C. elegans*, mutations in *unc-51* cause diverse axonal defects, including premature termination, abnormal trajectories, and extra axon branches [12]. Developing neurites accumulate abnormal vesicles and cisternae, suggesting underlying defects in membrane vesicle trafficking [18]. Intriguingly, Unc-51 directly interacts with Unc-14, a RUN domain protein, to regulate axonal elongation and guidance, and mutations in *unc-14* cause neurite growth and guidance defects that are very similar to those of *unc-51* [51]. Unc-14 regulates vesicle transport and localization by binding to Unc-16/JIP3/JSAP1, which is a cargo adaptor for the kinesin motor proteins [22]. Recent studies have shown that RUN domain proteins function as effectors of Rap and Rab GTPases in the control of membrane trafficking [54], highlighting the importance of vesicle trafficking in the regulation of axonal growth and guidance. Several studies have suggested that Unc-51 plays an essential role in the delivery of specific receptors for axonal guidance molecules [19], [20], [21], [23], [55]. Together with Unc-14, Unc-51 regulates the subcellular localization of the Netrin receptor/Unc-5 in *C. elegans* [23]. Thus, in *unc-14* and *unc-51* mutants, the Netrin receptor accumulates in neural cell bodies rather than at axonal termini, causing severe guidance defects in the DD/DV neurons [23]. Unc-51 also interacts with the kinesin-related Vab-8 protein, which regulates anterior-posterior migration of *C. elegans* mechanosensory neurons [55] through the regulation of another Netrin receptor Unc-40/Dcc and the Slit receptor Sax-3/Robo [19], [20], [21]. Vab-8 controls cell-surface expression of Sax-3/Robo in the growth cones of touch neurons through interaction with Unc-73/Trio, a guanine nucleotide exchange factor for Rac. Consequently, peptide-mediated interference with the Vab-8 and Unc-51 interaction in worm neurons blocked axonal outgrowth and posteriorly directed guidance [20].

In mouse, Unc51.1/Ulk1 is expressed in granule cells in the cerebellar cortex, and retroviral infection of immature granule cells with a dominant-negative Unc51.1 caused inhibition of neurite outgrowth both *in vitro* and *in vivo* [13]. Subsequent molecular studies showed that Unc51.1 binds to SynGAP and Syntenin [14], the latter of which, in turn, binds Rab5 GTPase to tether the Unc51.1/SynGAP/Rab5 complex to the vesicular membrane. Immunoelectron microscopy of granule cells provided evidence that Unc51.1 indeed associates with membrane vesicles. Moreover, SynGAP stimulates the GTPase activity of Rab5, and overexpression of SynGAP in cultured cerebellar granule neurons leads to truncated neurites and disorganized vesicular compartments [14]. This suggests that the Unc51.1-containing protein complex governs axonal elongation and pathfinding by modulating the Ras-like GTPase signaling pathway and the Rab5-mediated endocytic pathway in developing neurons.

The importance of Unc-51 in the regulation of vesicle trafficking is further supported by the finding that suppression of Unc-51 activity leads to increased neurite branch formation and shortened axons in cultured mouse dorsal root ganglia neurons [17]. Both Unc51.1 and Unc51.2 are localized to vesicular structures in growth cones in sensory axons, in which Unc51.1 promotes endocytosis of the neurotrophic tyrosine kinase receptor Ntrk1/TrkA through a non-clathrin mediated pathway, presumably through the interaction of Unc51.1 with SynGAP and Rab5 [17]. Moreover, Unc51.1 also interacts with the Golgi-associated ATPase enhancer of 16 kD (Gabarapl2/Gate-16), which is an essential factor for intra-Golgi transport [56]. Unc51.1 also interacts with the gamma-2 subunit of the GABA-A receptor associated protein (GABARAP), which is again involved in the regulation of receptor trafficking [56]. Together with our findings in the *Drosophila* brain, these studies highlight the functional importance of the Unc-51 family proteins in the endocytic processes that regulate diverse signaling events during axonal elongation, fasciculation, and guidance. Loss of the *Unc-51* activity is likely to perturb the trafficking of multiple types of post-Golgi vesicles and lead to severe disruption of the controlled delivery of essential axonal growth/guidance receptors to the different compartments of growing neurons. Elucidation of the exact molecular components that are involved in Unc-51-mediated regulation of vesicle transport is an important subject for future studies. We envisage that studies in *Drosophila* will continue to provide critical insights into the conserved molecular mechanisms of coordinate regulation of membrane vesicle trafficking and axon growth/guidance in developing neurons.

## Materials and Methods

### *Drosophila* strains

Fly strains were maintained on standard medium at 25°C. Construction of the *unc-51-*null mutants, *unc-51* ^3^ and *unc-51* ^25^, as well as *unc-51* ^WT^ and *unc-51* ^K38A^ transgenic flies has been described previously [16]. The following fly stocks were also used: *Klc* ^8e×94^ [44] (a gift from Joseph G. Gindhart), *Klc* ^c02312^ [45], UAS-*Fas II::YFP* [57] (a gift from Akinao Nose), *UAS-Dscam[TM1]::GFP* [47] (a gift from Tzumin Lee), *UAS-mCD8::DsRed* (a gift from Yuh Nung Jan), *GH146-GAL4* [34] (a gift from Reinhard F. Stocker), *UAS-Lamp1::GFP* [58] (a gift from Helmut Krämer), *OK107-GAL4* [59], [60], *201Y-GAL4* [61], *elav-GAL4* [62], *elav* ^c155^ [63], *tub-Gal80 FRT2A* [32], *elav* ^c155^ *UAS-mCD8::GFP hs-flp* [32], *UAS-tau::GFP* [30], *UAS-Nod::β-Gal* [50], *UAS-Syt1::eGFP* [64], *UAS-n-Syb::eGFP* [64], *UAS-Rab5::YFP* [65], *UAS-mito::GFP* [36], *UAS-hLC3::GFP* [39] and *UAS-Khc::eGFP* (Bloomington Stock Center). The *UAS-Klc::mRFP* stock was newly constructed for this work.

### MARCM mosaic analysis

Clones were generated using the MARCM method [32]. Egg collection was performed for 4 h on standard food at 25°C. Twenty-four hours after the end of egg collection, newly emerged larvae were heat shocked for 0.5 h (for the induction of MB clones) or 1 h (for the induction of AL-PN clones) at 37°C. Animals were fixed for examination at the wandering larval stage for the larval MB clones or at 2–3-day-old adult stage for the AL-PN clones.

The following genotypes were examined: (1) wild-type MBs: *elav* ^c155^ *hs-FLP UAS-mCD8::GFP/+; Gal80 FRT2A/FRT2A*; (2) *unc-51^−/−^* MBs: *elav* ^c155^ *hs-FLP UAS-mCD8::GFP/+; Gal80 FRT2A/unc-51* ^3^ *FRT2A*; (3) wild-type PNs: *hs-FLP UAS-mCD8::GFP/+; GH146-GAL4/+; Gal80 FRT2A/FRT2A*; (4) *unc-51^−/−^* PNs: *hs-FLP UAS-mCD8::GFP/+; GH146-GAL4/+; Gal80 FRT2A/unc-51* ^3^ *FRT2A*; (5) *Klc^−/−^* MBs: *elav* ^c155^ *hs-FLP UAS-mCD8::GFP/+; Gal80 FRT2A/Klc* ^8×94^ *FRT2A*.

### Immunohistochemistry

Immunological staining of larval and abult brains was performed as described in Kurusu et al. [28]. The primary antibodies used were: rabbit anti-SYT [66] (1∶1,000; a gift from Troy Littleton), guinea pig anti-Unc-51 (1∶100) [16], rabbit anti-*β*-Gal (1∶1000; Chemicon International), mouse anti-nc82 (1∶20; Developmental Studies Hybridoma Bank (DSHB)), mouse anti-Fas II (1∶5; DSHB), rat anti-N-Cadherin (1∶50; DSHB), mouse anti-Dac (1∶250; DSHB), rat anti-mCD8a (1∶100; Caltag), chicken anti-GFP (1∶500; Abcam), fluorescein isothiocyanate-conjugated horseradish peroxidase (1∶100; Jackson ImmunoResearch), Alexa 633-conjugated phalloidin (1∶100; Invitrogen). The secondary antibodies used were: Alexa 543-conjugated goat anti-rabbit, Alexa 488-conjugated goat anti-rabbit, Alexa 543-conjugated goat anti-guinea pig and Alexa 633-conjugated goat anti-mouse (1∶500; Molecular Probes). Confocal images were captured using a Zeiss LSM510 confocal microscope and processed with Adobe Photoshop.

## Supporting Information

Figure S1 **Localization of transport machinery proteins in** ***unc-51*** **mutant MBs.** (A, B) Localization of Khc as revealed with *UAS-Khc::eGFP*. Arrows indicate accumulations. Arrowhead in B2 indicates mislocalization at the proximal peduncle. (C, D) Localization of Klc as revealed with *UAS-Klc::mRFP*. (E, F) Localization of Tau as revealed with *UAS-tau::GFP*. (G, H) Localization of Futsch as revealed by anti-Futsch staining. The outline of the lobe is demarcated with yellow dashed lines in G3 and H3. Note that Futsch was detected in the cell bodies and the calyx but absent in lobes in both wild type and mutant MBs. (A, C, E, G) wild type. (B, D, F, H) *unc-51* ^25/25^ mutant. Late third instar larval stage. The calyx is demarcated with a yellow dashed circle. In A–F, marker expression was driven with *201Y-GAL4*. Scale bars, 10 µm. (I) Number of Khc::eGFP clogs in the lobes. Because of the background accumulation in the wild type lobes, only large clogs (>2 µm^2^) were counted. (J) Number of Khc::eGFP clogs in the cell bodies. (K) Number of Klc::mRFP clogs in the lobes. (L) Number of Klc::mRFP clogs in the cell bodies.
(TIF)

Figure S2 **Loss of** ***unc-51*** **causes axonal targeting defects in adult olfactory PNs.** (A–F) Axonal targeting phenotypes of wild-type (A, C, E) and *unc-51* ^3/3^ (B, D, F) clones in the adult brain. (A, B) anterodorsal neuroblast (adNb) clones. (C, D) lateral neuroblast (lNb) clones. (E, F) DL1 single cell clones. Clones were induced by an early first-instar heat shock and labeled with *UAS-mCD8::GFP* driven by *GH146-GAL4* (green). Neuropil was visualized with anti-nc82 (magenta). Yellow dashed lines demarcate the MB calyx (CX) and the lateral horn (LH). The yellow arrow in (D) indicates defasciculation and misrouting of the *unc-51* ^3/3^ axons. (G, H) Quantification of the axonal phenotypes of the DL1 single-cell clones. (G) The number of branches in the mushroom body calyx. Note the slightly increased branch number in *unc-51* ^3/3^ mutant (7.8; *n* = 9), compared with the wild type (6.4; *n* = 5). (H) The number of branches in the LH. No significant change was caused in branch numbers between the wild-type (5.6; *n* = 5) and the *unc-51* ^3/3^ mutant clones (5.0; *n* = 8). *P<0.05 by the Student's *t*-test. Scale bars, 30 µm in (A–D); 10 µm in (E–F).
(TIF)

Figure S3 **Loss of** ***unc-51*** **causes aberrant dendritic overextension of the olfactory PNs.** (A–F) Dendritic projection patterns of olfactory PNs in the adult brain. (A, B) anterodorsal neuroblast (adNb) clones. (C, D) lateral neuroblast (lNb) clones. (E, F) ventral neuroblast (vNb) clones. Wild-type (A, B, C) and *unc-51* ^3/3^ (B, D, F) clones. Clones were induced by an early first instar heat shock and labeled with *UAS-mCD8::GFP* driven by *GH146-GAL4*. Many of the *unc-51* mutant clones exhibited overextending dendrites. Arrow, long overextension; arrowhead, short overextension. (G) Quantification of the dendrite overextension phenotypes. Number of samples: wild type (*n* = 14), *unc-51* mutant (*n* = 22). Scale bar, 30 µm.
(TIF)

Figure S4 **Loss of** ***unc-51*** **causes moderate dendritic targeting defects in adult PNs.** (A–F) Dendritic targeting phenotypes of wild-type (A, C, E) and *unc-51* ^3/3^ (B, D, F) clones. Clones were induced by an early first-instar heat shock and labeled with *UAS-mCD8::GFP* driven by *GH146-GAL4* (green). Neuropil was visualized with anti-nc82 (magenta). Dendritic patterns of anterodorsal neuroblast (adNb) clones in the anterior (A, B), middle (C, D) and posterior (E, F) AL regions. VA1d, VA1lm, VA3, VM2, DM6, D, DL1 and VM7 are the landmark glomeruli normally innervated by *GH146*-positive ad-PNs (yellow letters). The VA2 glomerulus (white letters in A) is normally innervated by ad-PNs born in the embryonic stage, and is thus uninnervated in the wild-type clone. The DA1, DM5 and DM2 glomeruli (white letters) are normally innervated by the lateral PNs but not the ad-PNs. While dendritic targeting to the anterodorsal-type on-target glomeruli was normal, the lateral-type off-target glomeruli (DA1, DM5 and DM2) were occasionally innervated by the *unc-51* ^3/3^ adNb clones (yellow arrows in B and F). Scale bar, 10 µm. (K–N) Quantification of dendritic phenotypes of adNb clones. (K, M) Dendritic innervation of on-target glomeruli. Both wild-type and *unc-51* ^3/3^ clones exhibited complete innervation of all the on-target glomeruli. (L, N) Dendritic innervation of off-target glomeruli. The extent of innervation of the non-target glomeruli was classified into one of three groups: irregular, weak and no input. Sample sizes: wild type (*n* = 9) and *unc-51* ^3/3^ mutant (*n* = 10).
(TIF)

Figure S5 **Accumulation of endosomes and Golgi outposts in** ***unc-51*** **mutant MBs.** (A, B) Rab5::YFP. (C, D) Lamp1::GFP. (E, F) α-Man-II::eGFP. Late third instar larval stage. (A, C, E) wild type. (B, D, F) *unc-51* ^25/25^ mutant. MB expression was driven by *201Y-GAL4*. Arrows, aberrant accumulations of the marker proteins. Arrowhead, ectopic accumulation in the proximal peduncle. The calyx is demarcated with a yellow dashed circle. Scale bar, 10 µm. (G–I) Number of axonal clogs. (G) Rab5::YFP. (H) Lamp::GFP. (I) ManII::eGFP. The clog accumulations in the lobes were counted.
(TIF)

Figure S6 **Distribution of mitochondria and autophagosomes in wild-type and** ***unc-51*** **mutant MBs.** (A, B) mito::GFP. (C, D) hLC3::GFP. Late third instar larval stage. (A, C) wild type. (B, D) *unc-51* ^25/25^ mutant. MB expression was driven by *201Y-GAL4*. Arrow, aberrant accumulations. Arrowhead, ectopic accumulation in the proximal peduncle. The calyx is demarcated with a yellow dashed circle. Scale bar, 10 µm.
(TIF)

Figure S7 **Loss of** ***Klc*** **activity causes dendritic and axonal defects in MB neurons.** (A–D) Larval MB neuroblast clones generated by the MARCM technique. (A, C) Wild type. (B, D) *Klc* ^8e×94/8e×94^ mutant. (C, D) Peduncle sections. Note severe dendritic overextensions (arrows) and peduncle defasciculation (arrowhead) in the mutant. Clones were induced by a heat shock at the early first-instar stage and labeled with *mCD8::GFP* (green) driven by *elav* ^c155^ *-GAL4*. Mature axons were stained with anti-Fas II (magenta). (E–G) Quantification of the *Klc* mutant phenotype. (E) Quantification of the calyx (Cx) dendrite overextension phenotype. Sample sizes: wild type (*n* = 17), *Klc* ^8e×94/8e×94^ wild type (*n* = 7). (F) Quantification of peduncle defasciculation. Sample sizes: wild type (*n* = 8), *Klc* ^8e×94/8e×94^ mutant (*n* = 7). (G) Quantification of Fas II mislocalization in the proximal peduncle. Sample sizes: wild type (*n* = 11), *Klc* ^8e×94/8e×94^ mutant (*n* = 7), and *unc-51* ^−/−^ wild type (*n* = 10). Scale bars, 10 µm.
(TIF)

Figure S8 ***unc-51*** **does not interact with RPTPs in axonal fasciculation.** Larval MB lobes and peduncle sections (inset) from the late third instar stage. MBs were labeled with anti-Fas II (green) and anti N-Cadherin (magenta) (A, B, C, F, G), or phalloidin (magenta) (D, E). None of the double heterozygotes with the tested *RPTP* genes (*Lar*, *ptp69D* and *ptp10D*) showed abnormality in MBs, retaining wild-type like peduncle and lobes with a single core. Scale bars, 10 µm.
(TIF)

Figure S9 **Localization of Dscam and Nod in** ***unc-51*** **mutant MBs.** (A–F) Localization of Dscam as revealed with *UAS-Dscam[TM1]::GFP*. (G–L) Localization of Nod as revealed with *UAS-Nod::β-Gal*. Late third instar stage. MB neurons were labeled with *UAS-mCD8::DsRed* (A–F) or *UAS-mCD8::GFP* (G–L) in conjunction with *201Y-GAL4*. The arrowheads in (K) indicate Nod::β-Gal localization in calyx overextensions. Scale bar, 10 µm. (M, N) Quantification of the number of the Dscam::GFP clogs. (O, P) Quantification of the number of the Nod::β-Gal clogs. Clog accumulations in the lobes (M, O) or the cell bodies (N, P) were counted.
(TIF)
