# Supplementary figures and images for: Unc-51/ATG1 Controls Axonal and Dendritic Development via Kinesin-Mediated Vesicle Transport in the Drosophila Brain
Source: PLoS One. 2011 May 12;6(5):e19632. doi: 10.1371/journal.pone.0019632 (PMC3093397; doi:10.1371/journal.pone.0019632)

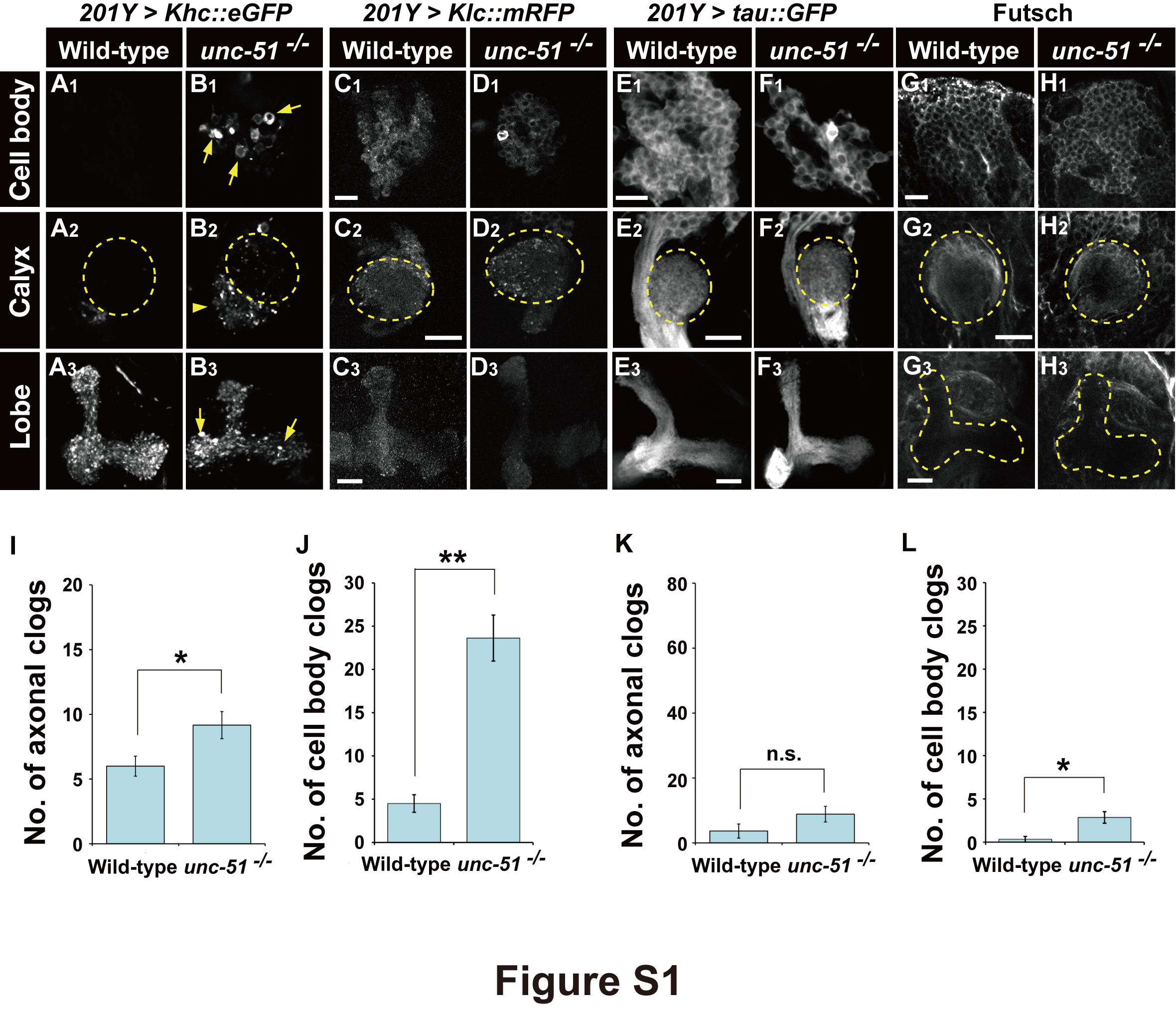

Supplement: Figure S1 — Localization of transport machinery proteins in unc-51 mutant MBs. (A, B) Localization of Khc as revealed with UAS-Khc::eGFP. Arrows indicate accumulations. Arrowhead in B2 indicates mislocalization at the proximal peduncle. (C, D) Localization of Klc as revealed with UAS-Klc::mRFP. (E, F) Localization of Tau as revealed with UAS-tau::GFP. (G, H) Localization of Futsch as revealed by anti-Futsch staining. The outline of the lobe is demarcated with yellow dashed lines in G3 and H3. Note that Futsch was detected in the cell bodies and the calyx but absent in lobes in both wild type and mutant MBs. (A, C, E, G) wild type. (B, D, F, H) unc-51 25/25 mutant. Late third instar larval stage. The calyx is demarcated with a yellow dashed circle. In A–F, marker expression was driven with 201Y-GAL4. Scale bars, 10 µm. (I) Number of Khc::eGFP clogs in the lobes. Because of the background accumulation in the wild type lobes, only large clogs (>2 µm2) were counted. (J) Number of Khc::eGFP clogs in the cell bodies. (K) Number of Klc::mRFP clogs in the lobes. (L) Number of Klc::mRFP clogs in the cell bodies. (TIF) [file pone.0019632.s001.tif]

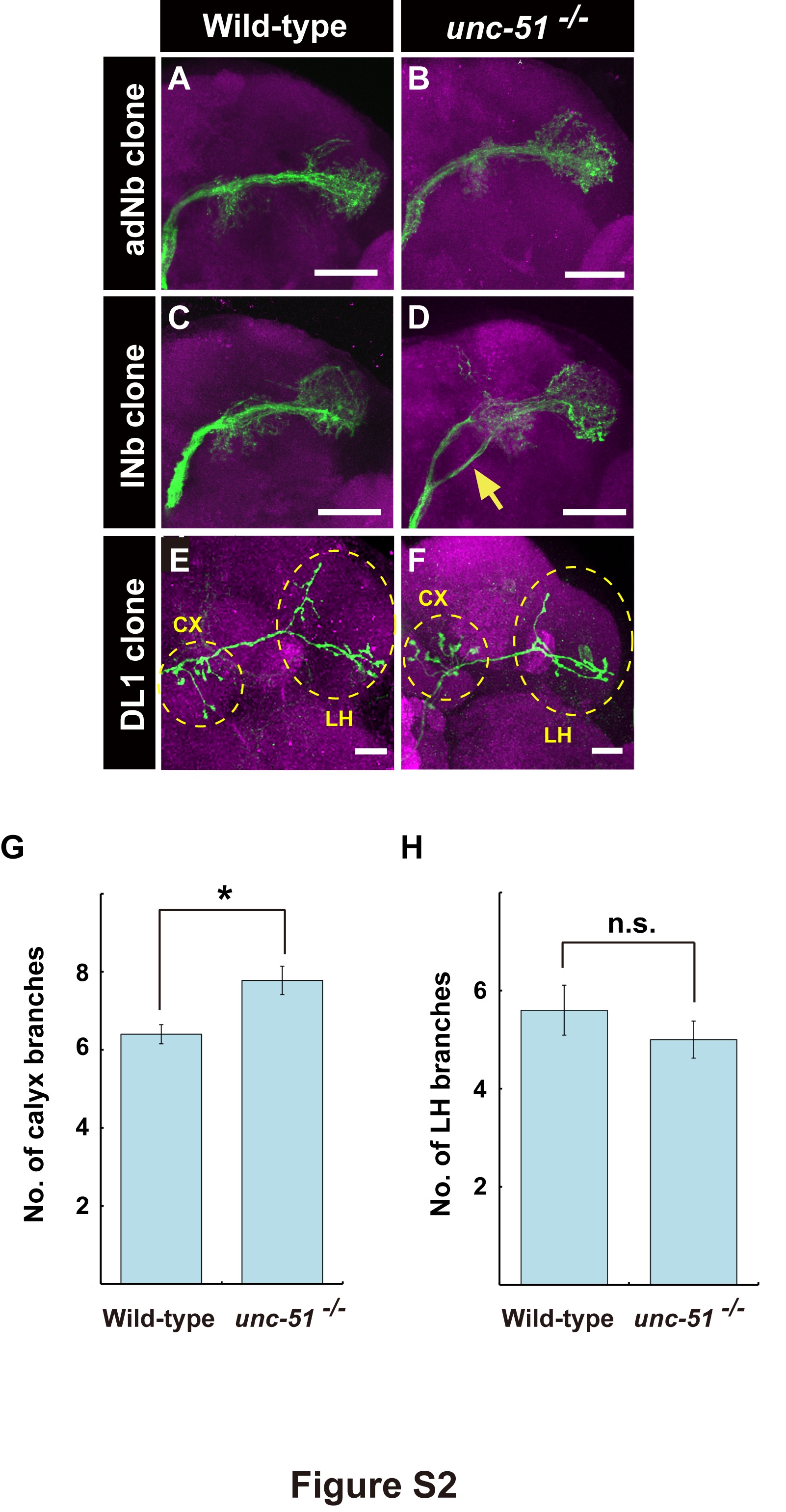

Supplement: Figure S2 — Loss of unc-51 causes axonal targeting defects in adult olfactory PNs. (A–F) Axonal targeting phenotypes of wild-type (A, C, E) and unc-51 3/3 (B, D, F) clones in the adult brain. (A, B) anterodorsal neuroblast (adNb) clones. (C, D) lateral neuroblast (lNb) clones. (E, F) DL1 single cell clones. Clones were induced by an early first-instar heat shock and labeled with UAS-mCD8::GFP driven by GH146-GAL4 (green). Neuropil was visualized with anti-nc82 (magenta). Yellow dashed lines demarcate the MB calyx (CX) and the lateral horn (LH). The yellow arrow in (D) indicates defasciculation and misrouting of the unc-51 3/3 axons. (G, H) Quantification of the axonal phenotypes of the DL1 single-cell clones. (G) The number of branches in the mushroom body calyx. Note the slightly increased branch number in unc-51 3/3 mutant (7.8; n = 9), compared with the wild type (6.4; n = 5). (H) The number of branches in the LH. No significant change was caused in branch numbers between the wild-type (5.6; n = 5) and the unc-51 3/3 mutant clones (5.0; n = 8). *P<0.05 by the Student's t-test. Scale bars, 30 µm in (A–D); 10 µm in (E–F). (TIF) [file pone.0019632.s002.tif]

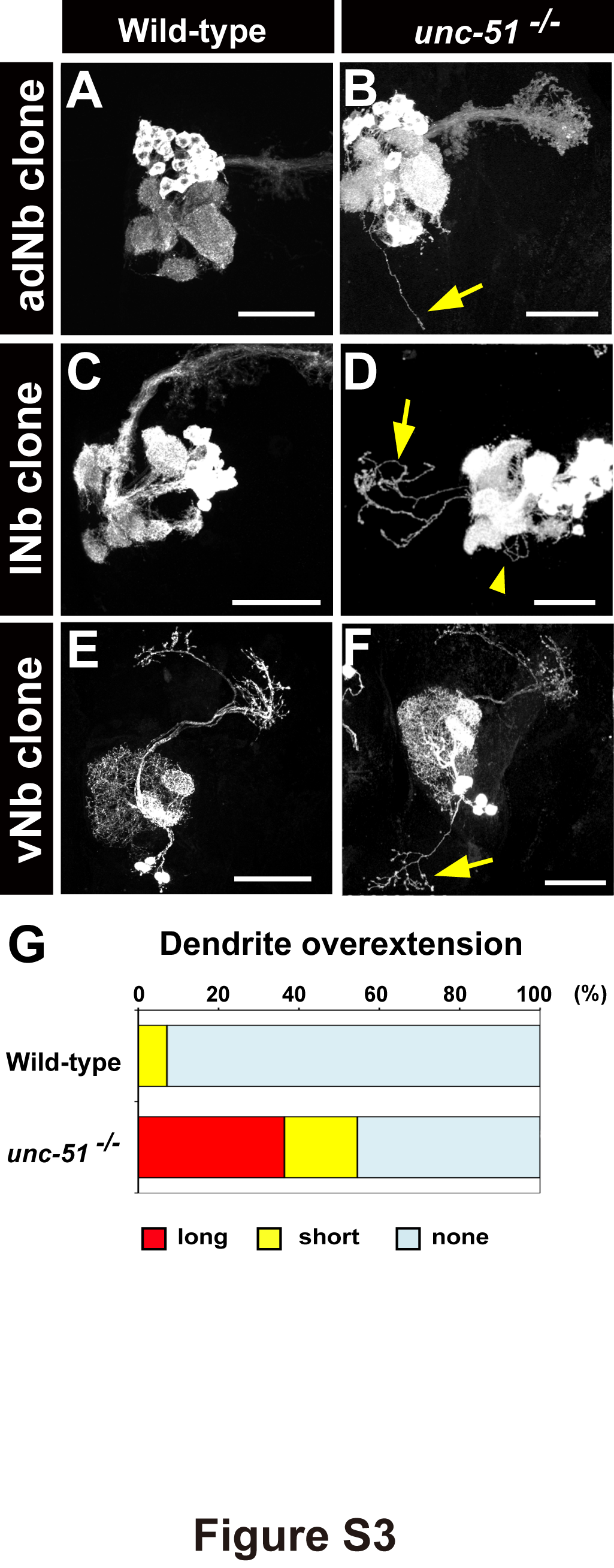

Supplement: Figure S3 — Loss of unc-51 causes aberrant dendritic overextension of the olfactory PNs. (A–F) Dendritic projection patterns of olfactory PNs in the adult brain. (A, B) anterodorsal neuroblast (adNb) clones. (C, D) lateral neuroblast (lNb) clones. (E, F) ventral neuroblast (vNb) clones. Wild-type (A, B, C) and unc-51 3/3 (B, D, F) clones. Clones were induced by an early first instar heat shock and labeled with UAS-mCD8::GFP driven by GH146-GAL4. Many of the unc-51 mutant clones exhibited overextending dendrites. Arrow, long overextension; arrowhead, short overextension. (G) Quantification of the dendrite overextension phenotypes. Number of samples: wild type (n = 14), unc-51 mutant (n = 22). Scale bar, 30 µm. (TIF) [file pone.0019632.s003.tif]

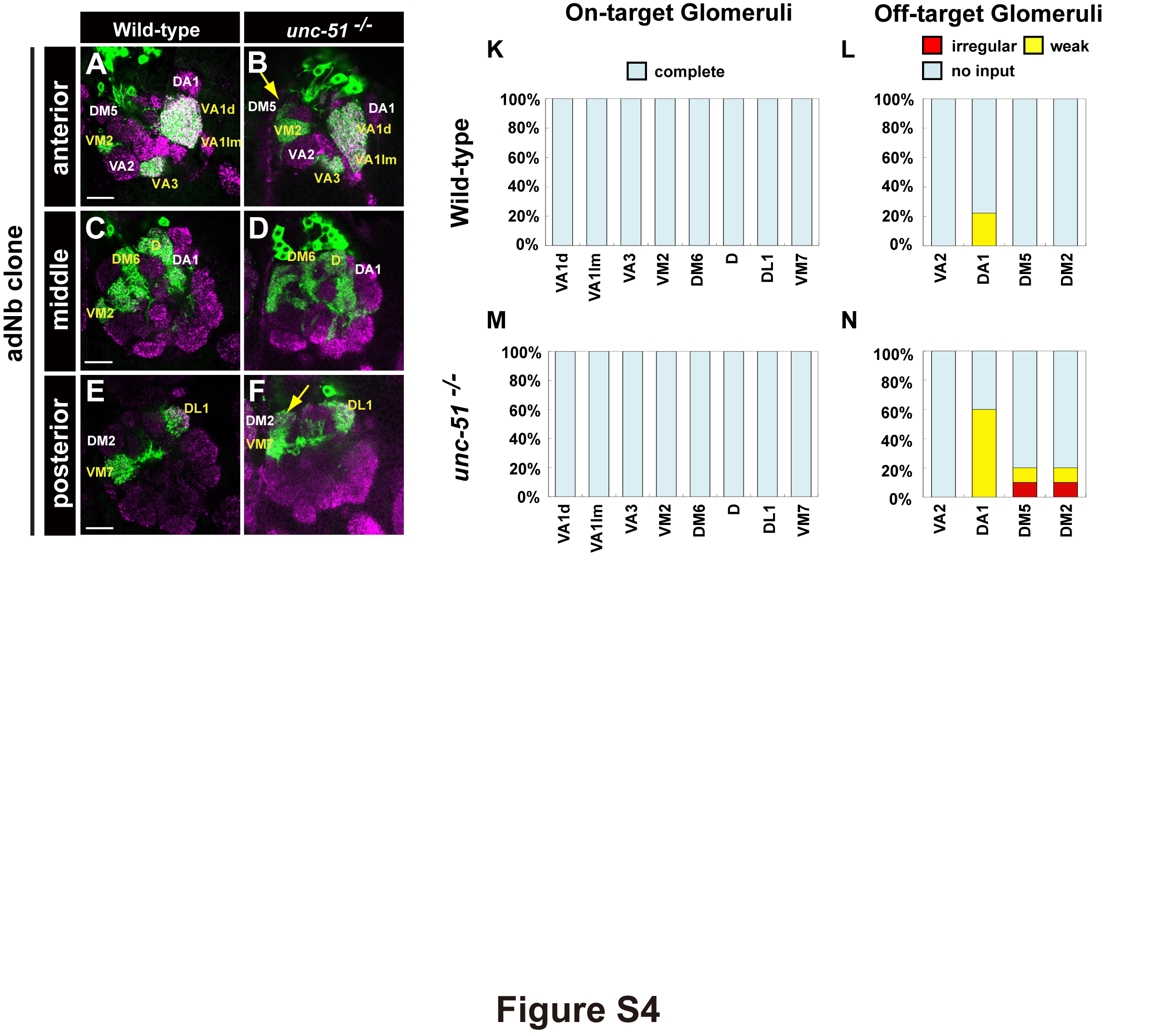

Supplement: Figure S4 — Loss of unc-51 causes moderate dendritic targeting defects in adult PNs. (A–F) Dendritic targeting phenotypes of wild-type (A, C, E) and unc-51 3/3 (B, D, F) clones. Clones were induced by an early first-instar heat shock and labeled with UAS-mCD8::GFP driven by GH146-GAL4 (green). Neuropil was visualized with anti-nc82 (magenta). Dendritic patterns of anterodorsal neuroblast (adNb) clones in the anterior (A, B), middle (C, D) and posterior (E, F) AL regions. VA1d, VA1lm, VA3, VM2, DM6, D, DL1 and VM7 are the landmark glomeruli normally innervated by GH146-positive ad-PNs (yellow letters). The VA2 glomerulus (white letters in A) is normally innervated by ad-PNs born in the embryonic stage, and is thus uninnervated in the wild-type clone. The DA1, DM5 and DM2 glomeruli (white letters) are normally innervated by the lateral PNs but not the ad-PNs. While dendritic targeting to the anterodorsal-type on-target glomeruli was normal, the lateral-type off-target glomeruli (DA1, DM5 and DM2) were occasionally innervated by the unc-51 3/3 adNb clones (yellow arrows in B and F). Scale bar, 10 µm. (K–N) Quantification of dendritic phenotypes of adNb clones. (K, M) Dendritic innervation of on-target glomeruli. Both wild-type and unc-51 3/3 clones exhibited complete innervation of all the on-target glomeruli. (L, N) Dendritic innervation of off-target glomeruli. The extent of innervation of the non-target glomeruli was classified into one of three groups: irregular, weak and no input. Sample sizes: wild type (n = 9) and unc-51 3/3 mutant (n = 10). (TIF) [file pone.0019632.s004.tif]

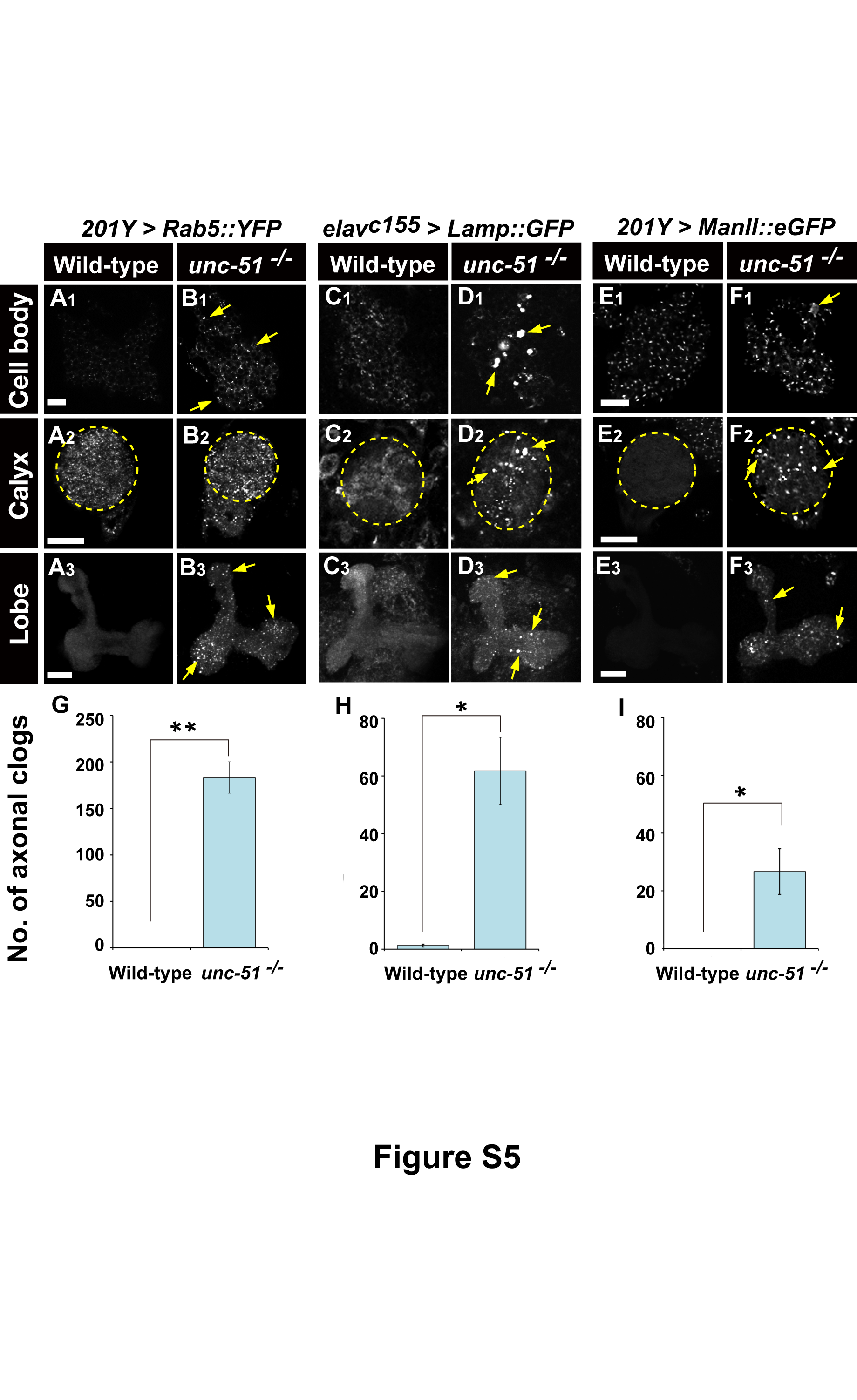

Supplement: Figure S5 — Accumulation of endosomes and Golgi outposts in unc-51 mutant MBs. (A, B) Rab5::YFP. (C, D) Lamp1::GFP. (E, F) α-Man-II::eGFP. Late third instar larval stage. (A, C, E) wild type. (B, D, F) unc-51 25/25 mutant. MB expression was driven by 201Y-GAL4. Arrows, aberrant accumulations of the marker proteins. Arrowhead, ectopic accumulation in the proximal peduncle. The calyx is demarcated with a yellow dashed circle. Scale bar, 10 µm. (G–I) Number of axonal clogs. (G) Rab5::YFP. (H) Lamp::GFP. (I) ManII::eGFP. The clog accumulations in the lobes were counted. (TIF) [file pone.0019632.s005.tif]

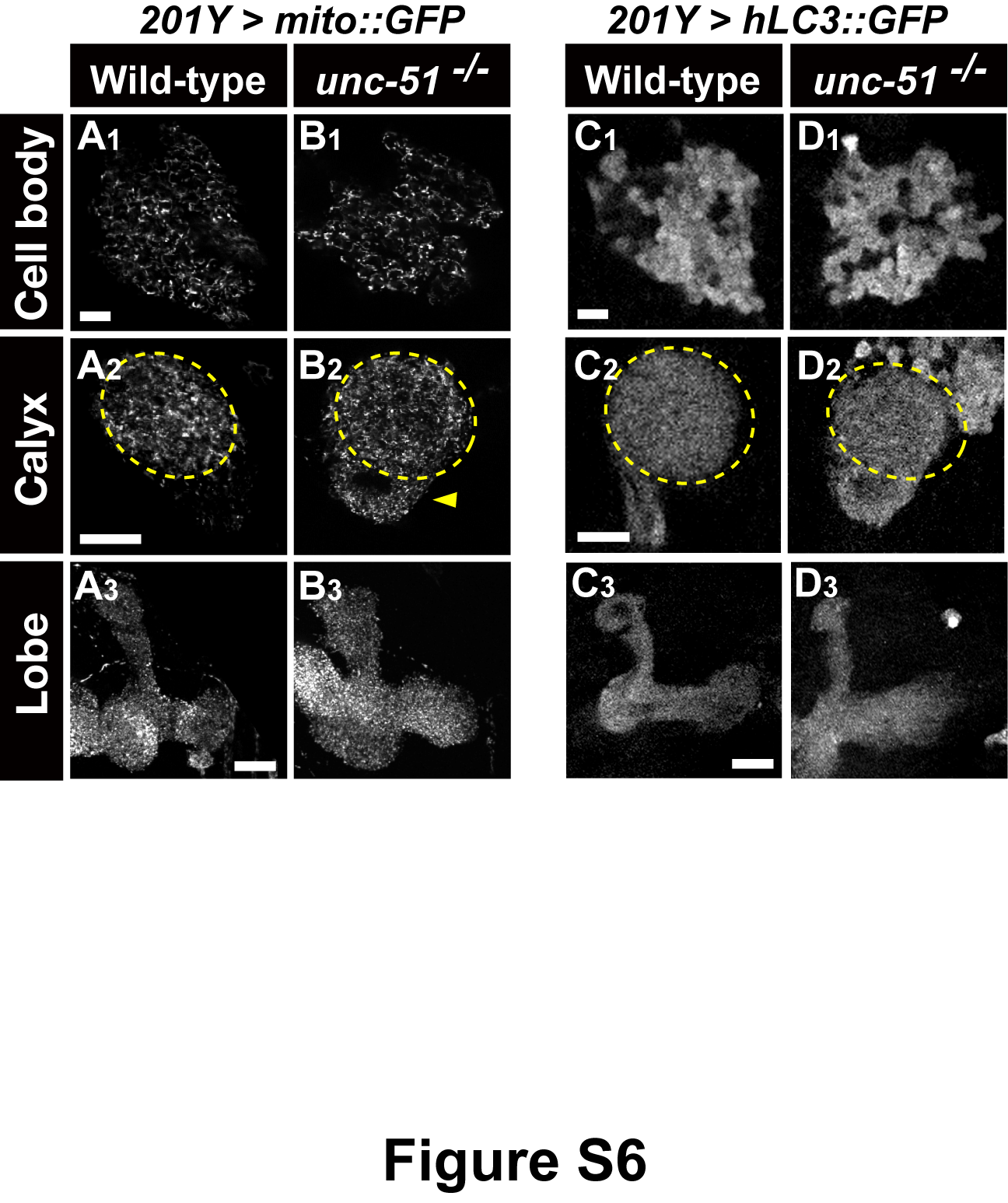

Supplement: Figure S6 — Distribution of mitochondria and autophagosomes in wild-type and unc-51 mutant MBs. (A, B) mito::GFP. (C, D) hLC3::GFP. Late third instar larval stage. (A, C) wild type. (B, D) unc-51 25/25 mutant. MB expression was driven by 201Y-GAL4. Arrow, aberrant accumulations. Arrowhead, ectopic accumulation in the proximal peduncle. The calyx is demarcated with a yellow dashed circle. Scale bar, 10 µm. (TIF) [file pone.0019632.s006.tif]

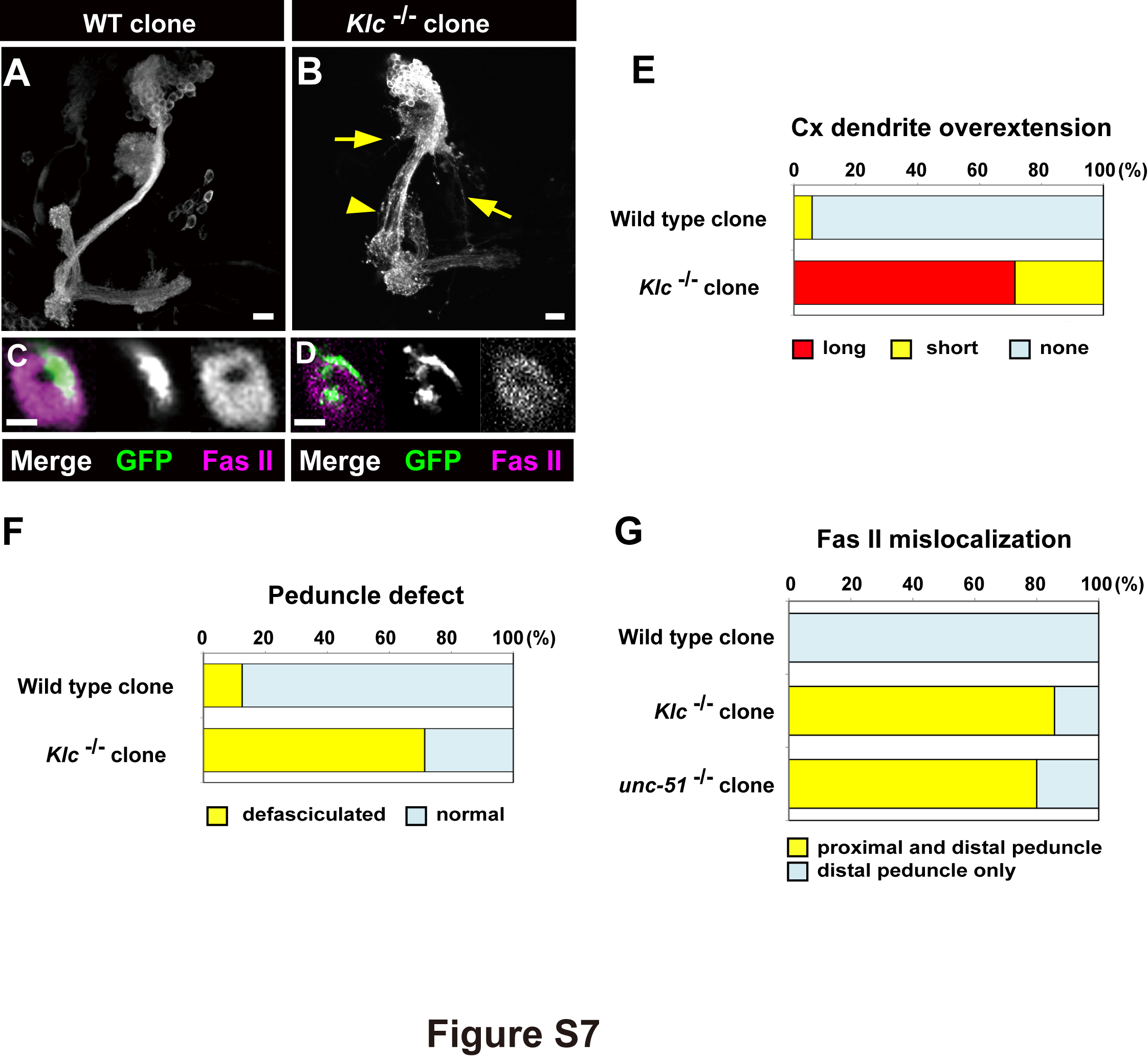

Supplement: Figure S7 — Loss of Klc activity causes dendritic and axonal defects in MB neurons. (A–D) Larval MB neuroblast clones generated by the MARCM technique. (A, C) Wild type. (B, D) Klc 8e×94/8e×94 mutant. (C, D) Peduncle sections. Note severe dendritic overextensions (arrows) and peduncle defasciculation (arrowhead) in the mutant. Clones were induced by a heat shock at the early first-instar stage and labeled with mCD8::GFP (green) driven by elav c155 -GAL4. Mature axons were stained with anti-Fas II (magenta). (E–G) Quantification of the Klc mutant phenotype. (E) Quantification of the calyx (Cx) dendrite overextension phenotype. Sample sizes: wild type (n = 17), Klc 8e×94/8e×94 wild type (n = 7). (F) Quantification of peduncle defasciculation. Sample sizes: wild type (n = 8), Klc 8e×94/8e×94 mutant (n = 7). (G) Quantification of Fas II mislocalization in the proximal peduncle. Sample sizes: wild type (n = 11), Klc 8e×94/8e×94 mutant (n = 7), and unc-51 −/− wild type (n = 10). Scale bars, 10 µm. (TIF) [file pone.0019632.s007.tif]

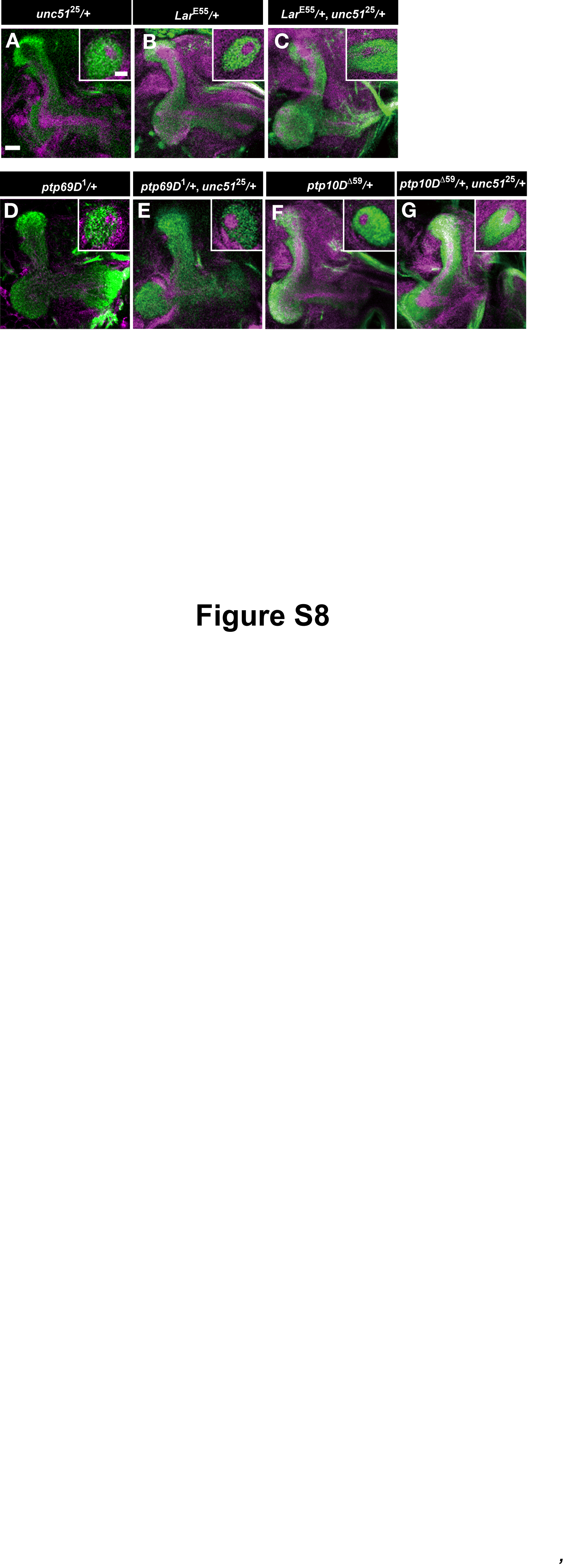

Supplement: Figure S8 — unc-51 does not interact with RPTPs in axonal fasciculation. Larval MB lobes and peduncle sections (inset) from the late third instar stage. MBs were labeled with anti-Fas II (green) and anti N-Cadherin (magenta) (A, B, C, F, G), or phalloidin (magenta) (D, E). None of the double heterozygotes with the tested RPTP genes (Lar, ptp69D and ptp10D) showed abnormality in MBs, retaining wild-type like peduncle and lobes with a single core. Scale bars, 10 µm. (TIF) [file pone.0019632.s008.tif]

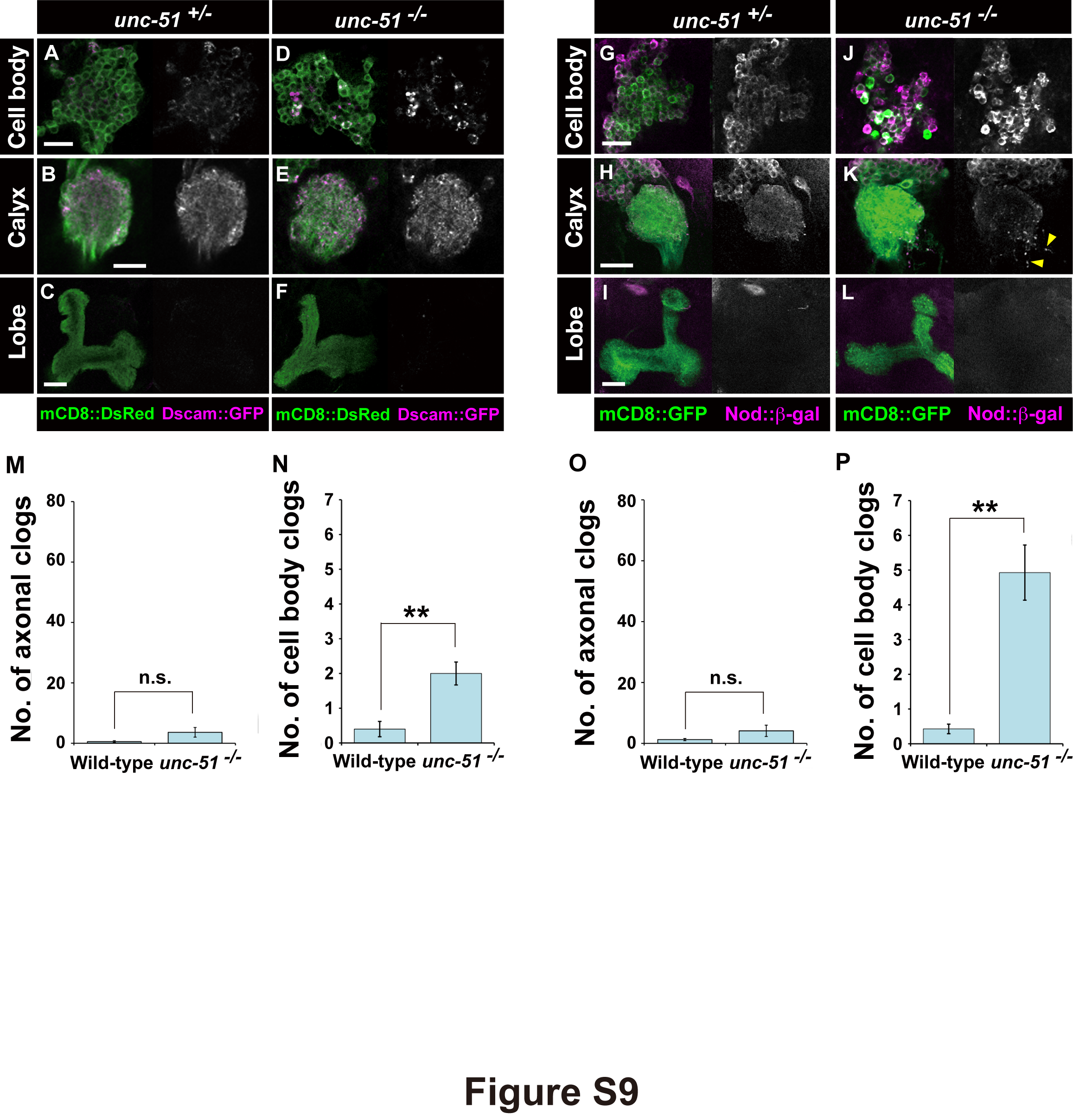

Supplement: Figure S9 — Localization of Dscam and Nod in unc-51 mutant MBs. (A–F) Localization of Dscam as revealed with UAS-Dscam[TM1]::GFP. (G–L) Localization of Nod as revealed with UAS-Nod::β-Gal. Late third instar stage. MB neurons were labeled with UAS-mCD8::DsRed (A–F) or UAS-mCD8::GFP (G–L) in conjunction with 201Y-GAL4. The arrowheads in (K) indicate Nod::β-Gal localization in calyx overextensions. Scale bar, 10 µm. (M, N) Quantification of the number of the Dscam::GFP clogs. (O, P) Quantification of the number of the Nod::β-Gal clogs. Clog accumulations in the lobes (M, O) or the cell bodies (N, P) were counted. (TIF) [file pone.0019632.s009.tif]
